# Inhibition of MMP2-PEX by a novel ester of dihydroxy cinnamic and linoleic acid from the seagrass *Cymodocea serrulata*

**DOI:** 10.1038/s41598-021-90845-9

**Published:** 2021-06-01

**Authors:** V. S. Christina, R. Lakshmi Sundaram, V. Sivamurugan, D. Thirumal Kumar, C. D. Mohanapriya, V. L. Shailaja, S. P. Thyagarajan, C. George Priya Doss, K. Mary Elizabeth Gnanambal

**Affiliations:** 1 Department of Biotechnology, Faculty of Biomedical Sciences and Technology, SRI RAMACHANDRA Institute of Higher Education and Research (SRIHER), Deemed to be University (DU), Porur, Chennai, Tamil Nadu 600 116 India; 2 Central Research Facility (CRF), SRI RAMACHANDRA Institute of Higher Education and Research (SRIHER), Deemed to be University (DU), Porur, Chennai, Tamil Nadu 600 116 India; 3 PG & Research Department of Chemistry, Pachaiyappa’s College, Chennai, Tamil Nadu 600 030 India; 4 Meenakshi Academy of Higher Education and Research, Chennai, Tamil Nadu 600 078 India; 5 Avinashilingam Institute for Home Science and Higher Education for Women (Deemed University), Coimbatore, Tamil Nadu 641 043 India; 6 Department of Integrative Biology, School of Biosciences and Technology, Vellore Institute of Technology, Vellore, Tamil Nadu 632014 India

**Keywords:** Cancer, Computational biology and bioinformatics, Drug discovery

## Abstract

Matrix metalloproteinases (MMPs) are pivotal for cancer cell migration and metastasis which are generally over-expressed in such cell types. Many drugs targeting MMPs do so by binding to the conserved catalytic domains and thus exhibit poor selectivity due to domain-similarities with other proteases. We report herein the binding of a novel compound [3-(*E*-3,4-dihydroxycinnamaoyloxyl)-2-hydroxypropyl 9Z, 12Z-octadeca-9, 12-dienoate; Mol. wt: 516.67 Da], (**C**_**1**_), isolated from a seagrass, *Cymodocea serrulata* to the unconserved hemopexin-like (PEX) domain of MMP2 (− 9.258 kcal/mol). MD simulations for 25 ns, suggest stable ligand**-**target binding. In addition, **C**_**1**_ killed an ovarian cancer cell line, PA1 at IC_50_: 5.8 μM (lesser than Doxorubicin: 8.6 µM) and formed micronuclei, apoptotic bodies and nucleoplasmic bridges whilst causing DNA laddering, S and G2/M phase dual arrests and MMP disturbance, suggesting intrinsic apoptosis. The molecule increased mRNA transcripts of BAX and BAD and down-regulated cell survival genes, Bcl-xL, Bcl-2, MMP2 and MMP9. The chemical and structural details of **C**_**1**_ were deduced through FT-IR, GC–MS, ESI–MS, ^1^H and ^13^C NMR [both 1D and 2D] spectra.

## Introduction

Molecules from seagrasses [Family: Hydrocharitaceae] not only display chemical diversity, but also demonstrate bioactivities with specificities^1–3^. Among the states in India, Tamil Nadu, located in the southeast coast, has extensive seagrass meadows comprising of 7 genera and 12 species. *Cymodocea serrulata* is one that grows profusely. Matrix Metalloproteinases (MMPs) are important in cancer cell migration and metastasis, as evident from previous reports^4,5^. Several molecules that target MMPs, fail to get elevated as potent drug candidates because they bind to the catalytic domains that are highly conserved, displaying poor selectivity and thus bind to other proteases, invariably yielding side effects^6,7^. Hence researchers are always “on-the-go” to find novel molecules that act on non-catalytic/unconserved regions of MMPs to gain specificities and minimize side effects. The present investigation deals with isolation and structure elucidation of a novel lipid class of molecule from the seagrass *C. serrulata*, which was identified as a glyceryl ester of 3,4-dihydroxy cinnamic and linoleic acid (assigned as **C**_**1**_ hereafter). The study also analyzes the possibilities of **C**_**1**_ to inhibit teratocarcinomic ovarian cell line, PA1. We understood that **C**_**1**_ binds to the less conserved hemopexin-like PEX domain of MMP2 with high specificities and compactness over a period of 25 ns, in silico. The study, at this juncture, provides background information on **C**_**1**_ to guide future research on this molecule to understand its implications on PEX-MMP2 and usage in cancer therapies.

## Methods and materials

### Seagrass collection and identification

Collection of the seagrass samples and proper identification protocols were followed as described in Global Seagrass Research Methods by Burdick and Kendrick^8^. Approximately 2.5 kg of the healthy wild seagrass with two or more shoots of *Cymodocea serrulata* (R.Br.) Asch. & Magnus*,* was handpicked from inter tidal areas (2–3 m deep) of Thonithurai (Lat: 11.48, Long: 79.76), Ramanathapuram, Southeast coast of India, by the research personnel by snorkeling. To ascertain concordance in the collection of samples, individual shoots were examined twice before picking and at least five sample sets were sent for identification, every time a collection was performed. This was done according to the sampling procedures listed in the above-mentioned manual. Appropriate permission for sample collection has been obtained from Dr. V. Veeragurunathan, Scientist, Central Salt and Marine Chemicals Research Institute (CSMCRI)-MARS Mandapam Camp, A Council of Scientific and Industrial Research (CSIR) (Organization), Mandapam, Ramanathanpuram, Tamil Nadu, India-623519. The samples were sent to Dr. V. Veeragurunathan, Scientist at CSIR-CSMCRI, Bhavnagar, Gujarat, India-364002 for identification. After identification, the samples were again sent to Dr. Patterson Edward, Director, Suganthi Devadason Marine Research Institute (SDMRI), Tuticorin, Tamil Nadu, India-628003 both for a re-confirmation and preservation as a voucher specimen for herbarium with the Ref No: [SDMRI/1/2014], which is accessible to the public for referencing purposes. Collection methods and appropriate permission for the particular species comply with the relevant national guidelines issued by National Biodiversity Authority (NBA) of India. The species does not come under threatened or near-to-extinction category as listed by National Biodiversity Authority, Ministry of Environment, Forest and Climate Change, Govt. of India (Ministry of Environment and Forest Notification, 2011, which is updated till date).

### Chemistry: preparation of the biological material for column chromatography and structure elucidation of C_1_

**Figure 1:**
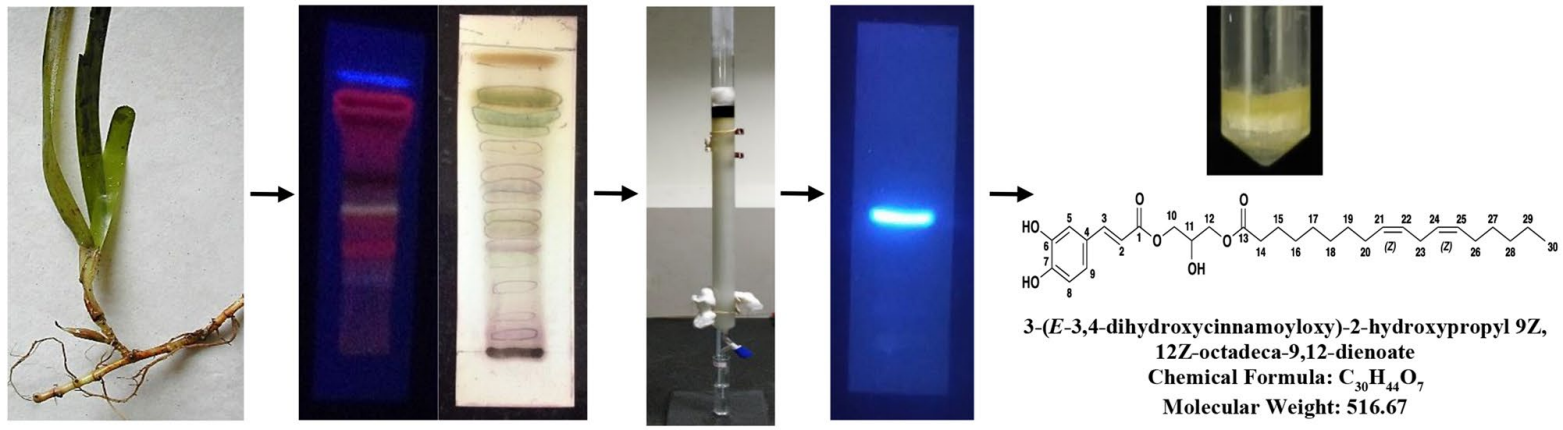
*Cymodocea serrulata*; Thin Layer Chromatograms of the active chloroform extract (hexane: ethyl acetate; 6:4) viewed at 356 nm and white light (derivatizing agent—methanol: sulphuric acid, 9:1); The active compound **C**_**1**_ was isolated using silica gel column chromatography (hexane: ethyl acetate; 90:10) and viewed at 356 nm (hexane: ethyl acetate; 9.5:0.5; R_*f*_ = 0.6); The structural and chemical details of **C**_**1**_.

Samples were cleaned with distilled water to remove debris and salt, and the cleaned leaves were shade-dried to remove moisture. The dried samples were pulverized to perform sequential extraction using organic solvents from low to high polarities: *n-*hexane, chloroform, ethyl acetate and methanol. Each solvent extract was accumulated individually and vacuum-distilled and the resultant residual extracts (Extract yield: Hexane: 1.6 g; Chloroform: 2.7 g; Ethyl acetate: 1.8 g and Methanol: 4.2 g) were used for preliminary bioassays. Out of these, chloroform extracts inhibited the growth of PA1 cell line at an IC_50_ of 10 µg mL^−1^, indicating its cytotoxic property (MTT assay^9^). Subsequently, the chloroform extracted residue was column-purified using silica gel (60–120 mesh), using appropriate combinations of hexane and ethyl acetate as eluents so that a polarity gradient could be achieved. A total of 830 fractions were obtained and one of the 46 fractions eluted from 90:10 [hexane: ethyl acetate] combination was found to be most active against PA1 cell lines at an IC_50_ of 3 µg mL^−1^ [5.8 μM]. The active fraction had R_*f*_ value of 0.6 when eluted with hexane: ethyl acetate in the ratio of 6:4 in TLC. The active compound [yield: 140 mg/500 g; 0.028% of dried seagrass biomass] was a yellowish-green colored semisolid viscous compound which fluoresced in natural day light and exhibited a bright blue fluorescence in long UV range (356 nm) and designated as **C**_**1**_ (Fig. 1)_._ After evaluating novelties in chemical structure of the compound, the isolation procedure was filed for patent [complete specification with 10 claims] under the Indian Jurisdiction and the same has been published in the Patent Office Journal: No. 46/2017 dated 17/11/2017 [A process for extraction of bio-active compounds exhibiting anticancer property from *Cymodocea serrulata* and product thereof; with Application No: 1293/CHE/2015 A].

The active compound (**C**_**1**_) was dissolved in chloroform and subjected to the following analyses: (1) UV–VIS Spectroscopy (Lambda-35 series Perkin Elmer spectrophotometer, MA, USA) [a spectrum was recorded from 200 to 800 nm]; (2) Fourier Transform-Infra Red (FT-IR) Spectroscopy (FT-IR spectrophotometer, Jasco 4000 series, Maryland, USA) [5 mg of the compound was compressed with 200 mg of KBr as pellets]; (3) Gas Chromatogram-Mass Spectrometry (Perkin Elmer,Clarus-600 series, GC/MS) [Column conditions: Phase Elite 35 MS (Capillary column, Agilent make, dimensions: length—30.0 m; nominal diameter—250.00 μm; internal flow—1.4 mL/min; Column oven Temp: 50–280 °C; hold for 10 °C)]; (4) Electrospray Ionization-Mass Spectrometry (ESI–MS; Shimadzu Liquid Chromatography-Mass Spectrometry (LCMS)-8040 Triple Quadrupole System coupled with UHPLC (NEXERA)) [MS-EI^+ve^ 70 eV; Ion source temperature: − 200 °C, Column conditions: Flow rate: 350 µL min^−1^; DL Temp: 250 °C; Heat block: 450 °C]; and, (5) Nuclear Magnetic Resonance Spectroscopy (Bruker Ultrashield 400 MHz, Avance III) for both ^1^H and ^13^C recorded for 1D and 2D NMR (COSY, DEPT-135, HMBC, HSQC)) [**C**_**1**_ dissolved in 0.6 mL of CDCl_3,_ tetramethylsilane (TMS) (0.03%) was used as internal standard and the chemical shifts of the protons signals were calculated from this]. All the data were correlated and interpreted to predict the structural and chemical details of **C**_**1**_.

### Biology: general procedures for culture, maintenance, treatment and staining of PA1 and non-cancerous Chinese Hamster Ovarian (CHO) cell lines

Human ovarian teratocarcinomic cell line PA1 and non-cancerous epithelial ovarian cells CHO were purchased from The National Centre for Cell Sciences (NCCS), Govt. of India, Pune. These cells were cultured in 25 cm^2^ tissue culture flasks (SPL Life Sciences, Korea), using Minimum Essential Medium Eagle (MEM) (HiMedia, Bombay) which contained 10% Fetal Bovine Serum (FBS) (Gibco, USA). Generally, 1% of 100 X antibiotic–antimycotic solutions in combinations [Streptomycin (10 mg), Penicillin (10,000 units) and Amphotericin B (25 μg)] dissolved in 1 mL of saline (0.9%) that was used to prevent bacterial and fungal contaminations. Cell maintenance: pH 7.2 achieved using 5% CO_2_ at 37 °C with 95% air and is usually trypsinized [1X Trypsin–EDTA (HiMedia)] thrice a week; restricted to 15 passages from the seed stock. Cells of both the batches were inoculated in 96 well plates so as to achieve 1 × 10^3^ number of cells per well a day before the commencement of treatment for attachment. The cells were treated with the extracts (data not shown), **C**_**1**_ (1–120 µM after optimization) and Doxorubicin (Adriamycin, Mol. wt: 579.98 Da, Selleckchem, UK) used as positive control at 1–10 µM based on previous papers. After 24 h, 100 µL (Stock: 5 mg/10 mL) of 3-(4,5-dimethylthiazol-2-yl)-2,5diphenyltetrazolium bromide (MTT) [HiMedia, Bombay] was added onto each of the wells and left undisturbed for 3 h. Formazan, as purple colored crystals, (if formed, is indicative of MTT dye reduction aided by the active mitochondrial dehydrogenases), were dissolved using 100 µL Dimethyl Sulfoxide (DMSO), filtered and the clear solution was optically measured at 570 nm in an EnSpire Multimode Plate reader (PerkinElmer, USA). From the values obtained, percentage of viable cells was calculated as (OD of the test/OD of the control) × 100 and from this the Inhibitory Concentration_50_ (IC_50_) values for the test samples (extracts/**C**_**1**_/Doxorubicin) for each of the cell types were calculated.

For observing morphologies of the cells treated with **C**_**1**_/Doxorubicin and untreated controls, both the cells types were seeded onto 6 well flat bottom tissue culture petridishes (1 × 10^5^ cells/mL) and allowed to adhere overnight. Thereafter, the cells were treated with the test samples at the respective IC_50_ values. The cells were in turn incubated for 24 h and washed with Phosphate Buffered Saline at pH 7.2 in order to be studied using an Eclipse inverted microscope (Nikon T*i* E series, Japan) in phase contrast mode (10×). Stocked from 10 μg of each of the stains in one mL PBS, 10 μL of Acridine Orange and then Propidium Iodide (AO/PI, Sigma, USA) was added to the same set of cells for visualizing in fluorescent mode (Filter: CFI60) using 10× objectives (E_x/_E_m_: AO: 500/526 and PI: 493/636 nm)^10^.

For the purpose of staining the nucleus, both the treated and untreated cells were washed with PBS and then 4% paraformaldehyde was added and left undisturbed for 10 min. at 30 °C for fixing and thereafter treated with 0.2% Triton X-100 dissolved in PBS for 10 min. at the same temperature to gain cell permeability. After this, 4,6-diamidino-2-phenylindole **(**DAPI, Sigma, USA) (0.5 μg/mL PBS) was added to the cells and incubated for 5 min. The stained cells were again observed (E_x/_E_m_: DAPI: 359/461)^11^. After verifying that the IC_50_ values for the compound was ~ 40 times higher in CHO than used for PA1, safety of **C**_**1**_ to the non-target cells was established (evidence on safety of **C**_**1**_ is also provided in the in silico results as well; however, more validation could be attained when used in a panel of non-cancerous cell lines).

### Assessment of C_1_ for genotoxic risk, DNA laddering capabilities, cellular migration and cell cycle progression inhibition, mitochondrial membrane potential and gene expression altering abilities in PA1 and CHO cells

Both the cells were seeded, maintained and treated with test samples (**C**_**1**_ and Doxorubicin) for 24 h using the same protocols listed above. The cells were trypsinized and 5 × 10^3^ number of cells was reseeded into 30 mm tissue culture petridishes with MEM medium containing supplements as mentioned previously, but with a difference. While culture initiation, 3 μg/mL of a cytokinesis blocker Cytochalasin-B [CytoB, MW: 479.617 g/mol; Cayman, Michigan, USA] was added to arrest cell growth after forming binucleated cells. After 36 h (upon standardization), the cells were washed with PBS, fixed with ice cold methanol: acetic acid [3:1] for 5 min. and air-dried. Cells were stained with 5% Giemsa (Sigma, USA) by flooding the petridishes for 15–20 min. and rinsed thrice in double distilled water, air-dried and focused at 20 X in an Axioscope A1 Biology Microscope (Zeiss, Germany) in bright field. Details such as X and Y coordinates, number of cells with micronucleus (MN), number of MN in the BN (binucleated) cells and cells with one, two, three, four etc., nucleus were taken note of. The images of the cells were captured using Isis fluorescence imaging platform, Metasystems. A total of 1000 BN cells were observed and the MN frequency was calculated as number of MN in BN cells scored among the total number of BN cells observed. Nuclear Division Index (NDI) provides a measure of the proliferative status/cell cycle kinetics of the viable cell fraction upon treatment with specific drugs to evaluate whether or not the drug of interest is genotoxic. It was calculated by scoring around 1000 numbers of viable cells and then grouped as cell populations with 1, 2, 3 or 4 nuclei, using the formula: NDI = M1 + 2 (M2) + 3 (M3) + 4 (M4)/N, where, M1–M4 is the number of cells with 1–4 nuclei respectively and *N* is the total number of viable cells scored (excluding necrotic and apoptotic cells)^12^.

For analyzing the inhibitory action of the drug to cellular migrations, cells were seeded in 6 well plates as described previously and grown upto 80% confluence. Gaps were created in the center of the plates using sterile 10 μL micropipette tips and the cells were given the regular drug treatment as mentioned previously. Cell migration was seen in an inverted microscope and the images were captured both at the start of the experiment (0th h) and at the end (24th h). The distance migrated was measured thrice using an *Image-Pro* Plus Software (Version 6.3, Media Cybernetics, USA) and the mean differences between the 0th and 24th h were calculated to show the degree of migrations achieved in the treated and non-treated groups^13^. The cells at this stage were trypsinized and added with cell lysis buffer [25 mM EDTA, 200 mM NaCl, 10 mg/mL proteinase K (Genei, Bengaluru) and 10% Sodium Dodecyl Sulphate (SDS)] and incubated at 37 °C overnight for extraction of DNA. Thereafter, phenol/chloroform/isoamylalcohol (25:24:1) was added and the extracted material was stored in 1 M Tris–HCl and 500 mM Molecular Biology grade EDTA buffer (Merck, USA) [pH 8] at − 20 °C for long storage. Sample checks were done by electrophoresing DNA on 1.5% agarose (Lonza, Basel) gels and staining with 1 mg/mL Ethidium Bromide to visualize the separated bands in UVP BioImaging Systems (CA).

Cells distributed in various cell cycle phases were investigated between **C**_**1**_-treated as well as untreated PA1 cell groups by quantifying the DNA of the PI-stained cells on a flow cytometer. After seeding, growth and treatment, the cells were harvested with ice cold PBS, re-suspended in a RNA-free solution containing sodium citrate, Triton X-100 and PI. Finally the cells were sorted based on the amount of the stained DNA in a MoFlo XDP Cell Sorter (Beckman Coulter, CA) at 488 nm capturing 15,000 events (a minimum of 10,000 events will suffice data acquisition). Percentage of live and dead cells at individual phases of the cell cycle was determined by Summit software (version 4.3.02) that comes with the machine^14^.

In order to analyze MMP shifts, if any, a cationic dye like Rhodamine 123 was used, which generally accumulates in the mitochondria of live cells because of the maintained electron potential and thus fluoresces higher than when dispersed in the cytoplasm of the dead cells where the mitochondrial membrane is lost. For this, a portion of the treated and untreated cells was washed twice with PBS and incubated with 5 μg/mL of Rhodamine 123 (Merck, USA) for 30 min. at 37 °C in the dark. Then the fluorophore in the cells was measured at 540 nm in a BD Accuri C6 Plus FACS machine (BD Biosciences, New Jersey) using FL1A filter and analyzed for fluorescent shifts in the treated cells by keeping the untreated cell set as reference, by employing Accuri C6 plus analysis software in the instrument. Shifts in MMP were thus calculated taking into account both the number of cells and the intensities of fluorescence^14^.

Apoptotic genes such as, BAX, BAD, FasL and FADDR and anti-apoptotic genes like, Bcl-xL, Bcl-2, MMP2 as well as MMP9 were evaluated for the expression pattern on real-time in **C**_**1**_ treated cells using untreated batches as controls. Total RNA was extracted from the cells of both the groups using RNAisoPlus reagent (TaKaRa, Japan) and thereafter the purity of the RNA was determined in a Nanodrop machine (Nanodrop 1000, ThermoScientific, USA) using the routine 260/ 280 nm ratio. As described in the manufacturer’s procedure, 2 μg of the isolated RNA was reverse-transcribed using RevertAid First Strand complementary DNA (cDNA) synthesis kit (ThermoFisher Scientific, USA) so that a double stranded cDNA is first produced. Primers were designed for all the nine genes studied in this work based on the literature extant and further confirmed at https://blast.ncbi.nlm.nih.gov (list supplied as Table S2) for the purpose of amplification. The obtained PCR product was mixed with 2× concentration of SYBR Green mix and 5 pmol of the specific primers (based on previous standardization) to make the final volume to a 20 μL. The order of steps followed in a quantitative PCR (*q*PCR) is followed as given here: 50 °C for 2 min., 95 °C for 10 min. for 40 cycles, 95 °C for 15 s and finally at 60 °C for 1 min. in a 7500/7500 Fast Real-Time PCR System (Applied Biosystems, Carlsbad, CA) and the fluorescence emitted was measured [E_x_/E_m_ for SYBR Green: 497/520 nm]. As a routine process, the resultant mRNA expression levels were so normalized with endogenous control, Glyceraldehyde3-phosphate dehydrogenase (GAPDH). All the following processes: i.Collection of data,  ii. Sequence detection and iii. Data analysis were performed in Relative Quantification Manager software (QM, V1.2) by Comparative threshold (Ct) method and fold change was calculated as 2^−ΔΔCT^ as described by Schmittgen and Livak^15^.

### Statistical tools used

All the experiments having quantifiable end-points were performed thrice and the same was averaged; standard errors were assigned wherever required. Student’s ‘t’ test was used to quantify the differences between the treated and untreated. For the *q*PCR analysis, a minimum of three independent technical and biological replications were carried out. Differences between the genetic profiles of the induced and non-induced batches were calculated and validated statistically using probability values (*p*-values).

### Bioinformatics: tools for analyses, target prediction, interaction with other proteins and preparation of C_1_ for molecular docking simulations

Absorption, Distribution, Metabolism and Excretion (ADMET) studies using **C**_**1**_ were done using SwissADME^16^, pkCSM^17^ and ProTox-II^18^ servers. Putative targets for **C**_**1**_ were predicted using online SwissTargetPrediction server^19^ and from the identified targets, the most fitting ones (Hemopexin-like repeat domain of Matrix metalloproteinases (MMPs) 2 and 9) were chosen for further testing. The interacting proteins with these targets were retrieved from STRING database^20^. Thereafter two-Dimensional structure (2D) of the molecule was drawn and converted into an energy-minimized three-dimensional (3D) structure using the online Corina server and default settings (OPLS-2005) of Schrodinger LigPrep (LigPrep, Schrödinger, LLC, New York, NY, 2018) were used in optimizing the compound. LigPrep helps in assigning appropriate bonds and torsions of the compound and maintains the chirality, stereoisomers and ring conformations of the compound. The 3D structure of MMP2 (72 kDa type-IV collagenase) and MMP9 were retrieved from Protein Databases (PDB) with IDs, 1CK7 and 5UE3, respectively. The structures were further refined using the maestro software package and prime. Water molecules were removed from the protein molecules and ‘refinement only’ option of the protein preparation module was chosen to attain brief relaxation. Energy minimization was performed using default OPLS-2005 force field to alleviate steric clashes and active sites of the proteins were taken from previous reports^21,22^. Finally **C**_**1**_ was docked with MMP2 and MMP9 using the ‘‘extra precision’’ (XP) mode of Glide program (Glide, Schrödinger, LLC, New York, NY, 2018). Binding poses and surrounding amino acids were visualized using PyMOL and Schrodinger Maestro. These docked complexes were further subjected to Molecular Dynamics (MD) Simulations.

MD was performed using GROMACS package and GROMOS force-field versions 54A7 was used to parameterize the protein and ATB server was used to parameterize the ligand^23,24^. The complexed molecules were immersed with Simple Point Charge (SPC) water molecules in a cubical box and energy minimization was performed for 1000 steps as the maximum, to avoid steric clashes within the protein. Equilibrations were performed with NVT and NPT ensembles for 50,000 steps to equilibrate the system before a molecular dynamics simulation was performed. Finally, MD Simulations were performed for 25 ns and the trajectories were analyzed using embedded packages of GROMACS with the graphs plotted using XMGRACE software.

## Results

### Chemistry

All the data of spectral analyses of **C**_**1**_ are supplied as Figs. S1–S5. The chemical structure of **C**_**1**_ was elucidated based on the analyses reports from ^1^H and ^13^C NMR, GC–MS, ESI–MS and the compound is predicted to be an ester of 3,4-dihydroxy cinnamic and linoleic acid with glycerol and spectral data summarized in Table 1.

**Table 1:** ^1^H and ^13^C NMR assignments for **C**_**1**_ (CDCl_3_, 400 MHz).

H	Chemical shift (ppm) (400 MHz)	Assignment	C	Chemical shift (ppm) (100 MHz)	Assignment
–	–	–	**C1**	171.9	Ester, >C=O
**H2**	6.66–6.62 (d, 16 Hz, 1H)	=C–**H**	**C2**	114.1	=**C**-H
**H3**	7.59–7.55 (d, 16 Hz, 1H)	=C–**H**	**C3**	147.0	=**C**-H
–	–	–	**C4**	113.0	Ar-C
**H5**	7.42 (s) (1H)	Ar-**H**	**C5**	119.0	Ar-C
–	–	–	**C6**	143.0	Ar-C
–	–	–	**C7**	145.0	Ar-C
**H8**	7.38–7.36 (d, 8 Hz, 1H)	Ar-**H**	**C8**	116.0	Ar-C
**H9**	7.17–7.15 (d, 8 Hz, 1H)	Ar-**H**	**C9**	123.0	Ar-C
**H10 & H12**	4.35–4.31 (m, J = 4–6 Hz, 4H)	glyc C-**H**_**2**_	**C10 & C12**	62.1	glyc-**C**H_2_
**H11**	4.30–4.10 (m, J = 10 Hz, 1H)	glyc C-**H**	**C11**	64.9	glyc-**C**H
–	–	–	**C13**	173.3	Ester, >C=O
**H14**	2.40–2.32 (t, 7 Hz, 2H)	–C**H**_**2**_–C=O	**C14**	37.4	**C**H_2_–C=O
**H20 & H26**	2.08–2.06 (m, 8 Hz, 4H)	=CH–C**H**_2_	**C20 & C26**	27.2	CH=CH–**C**H_2_-
**H21, H22, H24 & H25**	5.40–5.37 (m, 6.4 Hz, 4H)	C**H**=C**H**–CH_2_–C**H**=C**H**–	**C21 & C25 C22 & C24**	130.0 127.9	–**C**H=**C**H–CH_2_–**C**H=**C**H–
**H23**	4.30–4.10 (m, 6–8 Hz, 2H)	CH=CH–C**H**_**2**_–CH=CH–	**C23**	24.8	–CH=CH–**C**H_2_–CH=CH–
**H15, H16, H19, H27 & H28**	1.45–1.28 (br, 10H)	Alkyl-C**H**_2_	**C15, C19, C27 & C28**	27.2, 26.4, 25.0, 24.8, 22.7	Alkyl-**C**H_2_
**H17, H18, H29 & H30**	0.93–0.87 (br, 9H)	Alkyl-C**H**_2_	**C17, C18, C29 & C30**	20.1, 19.7, 14.4, 14.1	Alkyl-**C**H_2_

The aromatic C_5_-H appears as a singlet at 7.42 ppm and that of C_8_ and C_9_-H protons as doublet between 7.32–7.33 ppm and 7.15–7.17 ppm respectively, with J value of 8 Hz, indicating *cis* coupling between the two aromatic protons. The alkenyl protons of C_3_ and C_2_ of caffeic acid moiety appearing as doublet in the range of 7.59–7.55 and 6.66–6.62 ppm respectively with coupling constant J value of 16 Hz is indicative of *trans* coupling between C_2_ and C_3_ of α and β unsaturated –C=C– in caffeic acid moiety. It is clear from ^13^C-NMR, that the ester carbonyl carbon (C_1_) of cinnamoyl group appeared at 171.9 ppm and the presence of phenolic –OH at C_6_ and C_7_ positions is shown as a broad singlet in the range of 5.37–5.40 ppm that finally merges with C_21_, C_22_, C_24_ and C_25_ alkenyl protons of linoleic acid. The alkenyl (C_2_ and C_3_) carbon appeared at 147.0 and 114.1 ppm. The aromatic carbons (C_6_ and C_7_) attached with phenolic –OH appeared at 143 and 145 ppm, whilst that of other aromatic carbons (C_5_, C_8_ and C_9_) at 119, 116 and 123 ppm respectively. Thus, based on the above observation, the presence of phenolic –OH groups, alkenyl protons and aromatic protons suggests the presence of 3,4-dihydroxy cinnamoyl moiety (*trans*-caffeic acid moiety) and confirms that the carbonyl group is an ester that is connected to a glycerol moiety. Presence of linoleic acid is ascertained by C_21_ and C_22_-alkenyl and C_24_ and C_25_ alkenyl protons (–C**H**=C**H**–CH_2_–C**H**=C**H**–) and all the alkenyl protons appearing as multiplet at 5.40–5.30 ppm with value of 6.4 Hz is due to the *cis* coupling of C_21_ and C_22_ alkenyl protons with that of allyl of CH_2_ at C_20_ and C_23_ carbon atoms and those of C_24_ and C_25_ alkenyl protons with allylic CH_2_ of C_23_ and C_26_. Allylic protons of C_23_-CH_2_ appeared as multiplets at 2.85–2.78 ppm and that of acyl protons (C_14_-CH_2_) which are attached to ester carbonyl appeared as a triplet at 2.65–2.62 ppm. Allylic protons of C_20_-CH_2_ and that of C_26_-CH_2_ showed as multiplets between 2.39 and 2.32 ppm. Alkyl protons of CH_2_ in linoleic acid attached to C_20_ and C_26_ all appeared as multiplets at 2.07–2.06 ppm and there was occurrence of alkyl protons at 1.41–1.28 ppm and 0.92–0.86 ppm as well.

In ^13^C NMR, the ester carbonyl group showing up at 173.13 ppm is due to linoleic acid attached to glycerol and alkenyl carbons (C_21_, C_22_, C_24_ and C_25_) appeared at 130.0 and 127.9 ppm. The α-CH_2_ carbon (C_14_) of linoleic acid attached to ester carbonyl appeared at 37.4 ppm and allylic carbon (C_23_) (–CH=CH–**C**H_2_–CH=CH–) attached to C_22_ and C_24_ at 24.8 ppm and another allylic carbon (C_20_ and C_26_) (–**C**H_2_–CH=CH–) attached to C_21_ and C_25_ at 27.2 ppm. However, alkyl carbons (–**C**H_2_–) of linoleic acid appeared between 15.0 and 25.0 ppm. C_10_-CH_2_ and C_12_-CH_2_ of glycerol appeared as doublet at 4.34–4.33 ppm with J value of 4.4 Hz, can be attributed to the vicinal coupling. J value 9.6 Hz could be due to *cis* coupling with methine protons (CH) of C_11_. The C_11_ methine protons appeared as multiplets at 4.13–4.10 ppm with J value of 4–6.8 Hz, which could be due to *cis* coupling with C_10_ and C_12_-CH_2_ protons of glycerol. In ^13^C NMR, C_10_ and C_12_-methylene carbon of glycerol appeared as a single peak at 62.10 ppm and that of C_11_-methine carbon at 64.9 ppm. Since the C_10_ and C_12_ of glycerol appeared as a single peak in ^13^C NMR, **C**_**1**_ could be of symmetrical nature, and 3,4-dihydroxy cinnamic acid and linoleic acid could be attached to C_10_ and C_12_ respectively; thus, C_11_ carbon is free from substitution. Therefore, based on the ^1^H and ^13^C NMR interpretation, the presence of two ester carbonyl carbons together with aromatic and alkenyl carbons revealed the presence of 3,4-dihydroxy cinnamoyl group and linoleic acid attached to glycerol. Hence, the isolated compound **C**_**1**_ could be an ester of 3,4-dihydroxy cinnamoyl group (*trans*-caffeic acid) and glycerol.

Gas Chromatogram of **C**_**1**_ showed two distinctive broad peaks, one at 6.57 and the other at 6.66 min. There is suspicion that the same fragments show up at 10.50, 15.10, 19.50 and 23.40 min as well, which was confirmed upon comparison with mass spectral analysis. Because the mass spectra showed similar fragmentation pattern as indicated by peaks appearing at 6.57 and 6.66 min, the results of MS of these two peaks (as shown in gas chromatogram) are discussed herein. Mass spectral analysis of the GC peak appeared at 6.57 min clearly indicates the presence of an unsaturated C_18_ fatty acid containing double bond in the alkyl chain. Mass spectrum showing a peak at *m/z* at 254 is due to the fragmentation of *trans*-3,4-dihydroxy cinnamic acid with glycerol ester and on the other hand, linoleic acid lose an acryloyl group (*m/z* 57), to give a signal at *m/z* 207. After the loss of methylene group (–CH_2_–), the peak appearing at *m/z* 195 confirms the presence of linoleic acid. In addition, molecular fragments at *m/z* 195, 180, 166, 152 in regular intervals by losing *m/z* 14 from CH_2_ radicals and the loss of propylene (CH_2_=CH–CH_2_·) appearing at *m/z* 41, both, are indicative of the presence of unsaturated fatty acid. Now the mass spectral analysis of the GC peak appeared at 6.66 min is discussed herein (Fig. S6). The peaks are similar to the fragmentation as obtained in Fig. S6A, due to the presence of linoleic acid as interpreted above. As observed in the above discussion, the mass spectrum followed similar mass pattern with regular loosing *m/z* 14 which indicates the loss of methylene (–CH_2_–) group from long alkyl chain. Also, mass fragmentation follows the general formula C_n_H_2n,_ which is suggesting that the molecule contains unsaturated fatty acid. ESI–MS of **C**_**1**_ shows that the sample is a single compound and in which, m/z peak at 515 is due to the presence of **C**_**1**_, whereas the peak at m/z 532 may be due to the presence of hydrated form of the **C**_**1**_. Based on the results concluded from FT-IR, ^1^H NMR, ^13^C NMR, GC–MS and ESI–MS spectral analyses, the compound is an ester of *trans*-3,4-dihydroxy cinnamic acid and linoleic acid connected to glycerol [3-(*E*-3,4-dihydroxycinnamaoyloxyl)-2-hydroxypropyl 9Z, 12Z-octadeca-9, 12-dienoate], with a mol. wt of 516.67 Da (Fig. 1). The chemical structure of the novel compound with all the spectral details is submitted publicly in the PubChem database [URL: https://pubchem.ncbi.nlm.nih.gov/substance/404333222 and https://pubchem.ncbi.nlm.nih.gov/compound/145864801] with the Substance Identification Number (SID): 404333222 and Compound Identification Number (CID): 145864801.

### Biology

**Figure 2:**
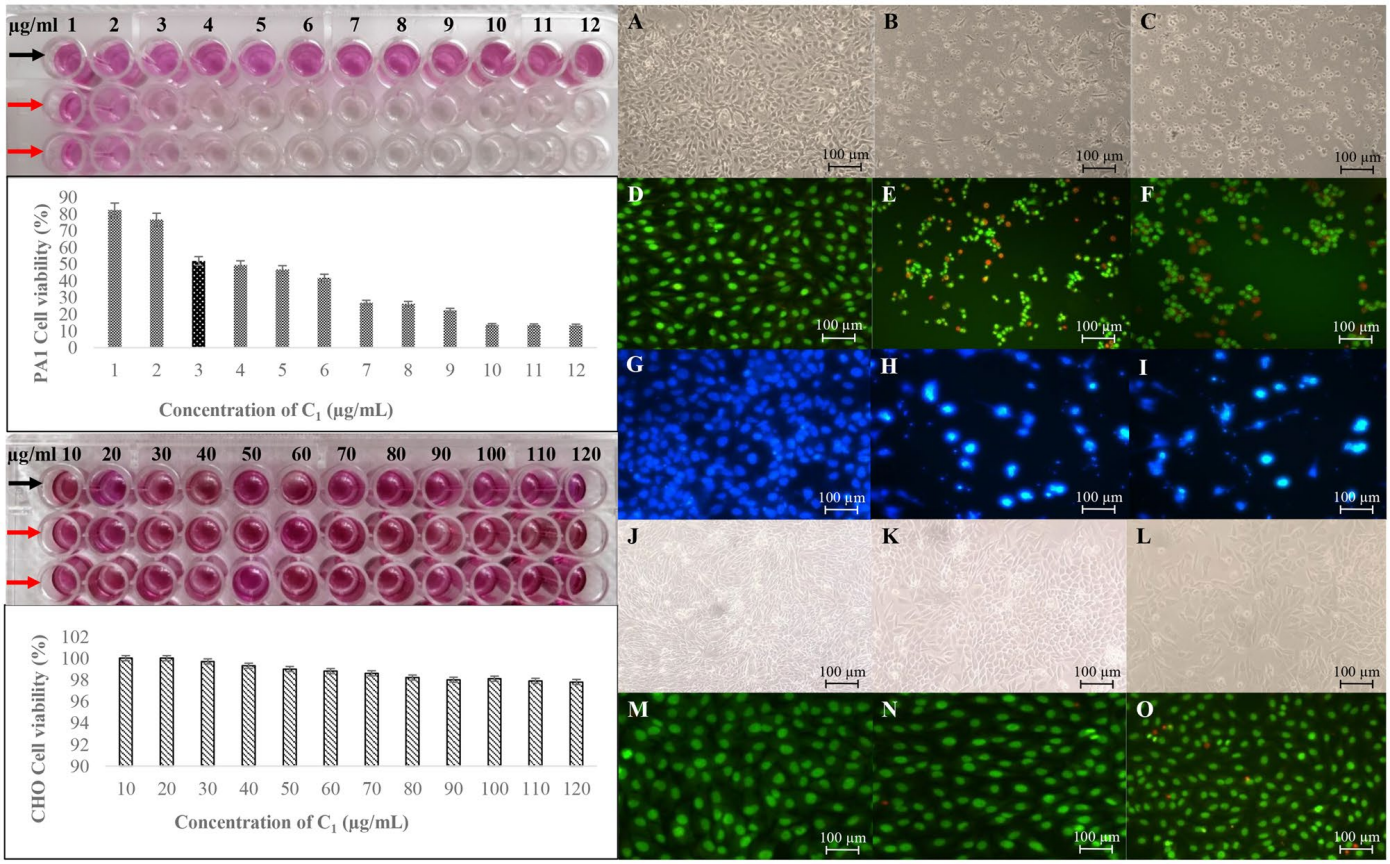
Cell viability assayed by formation of formazan crystals for the ovarian cancer (PA1) and normal Chinese Hamster Ovary (CHO) cell lines treated with **C**_**1**_ (red arrowheads and control indicated with black arrowheads) [left panel up and down respectively] with their IC_50_ values graphed right below. The compound **C**_**1**_ does not inhibit CHO cell lines even at 40 folds the IC_50_ concentration of PA1 cells (3 µg/mL [5.8 µM]). The right panel has micrographs of PA1 untreated cells [phase contrast (**A**), Acridine Orange/Propidium Iodide (AO/PI) double-stained (**D**) and 4′,6-diamidino-2-phenylindole (DAPI) stained (**G**)]. (**B**,**E**,**H)** are respectively for **C**_**1**_ treated cells, whilst (**C**,**F**,**I**) are Doxorubicin (IC_50_ 5 µg/mL [8.6 µM]) treated cells in the same sequence. (**J**) (Phase contrast) and (**M**) (AO/PI double-stained) represent CHO untreated cells, (**K**) and (**N**) are **C**_**1**_ treated while (**L**) and (**O**) are the Doxorubicin treated ones in the same order.

The outcomes of IC_50_ determinations for various sequential extracts of the plant material, *Cymodocea serrulata,* in PA1 ovarian cancer cell lines indicate that chloroform extracts possessed higher efficacy (IC_50_: 10 μg/mL) followed by *n*-hexane (IC_50_: 20 μg/mL), ethyl acetate (IC_50_: 25 μg/mL) and methanol (IC_50_: 40 μg/mL). Chloroform extract, based on activity-guided fractionation, yielded **C**_**1**_ which possessed least IC_50_ values to PA1 cells (5.8 μM). On the contrary, **C**_**1**_ does not kill even 5% of non-cancerous CHO cell lines (even at 232.25 μM), that is, more than 40 times the IC_50_ used for PA1 cell lines, indicating its safety to normal cells. Cell types, both PA1 and CHO treated with **C**_**1**_ and Doxorubicin (IC_50_: 8.6 µM) are shown as phase contrast micrographs to observe cellular morphologies. Fluorescent dyed pictures with AO/PI and DAPI to understand the nuclear changes as against their respective untreated controls are also shown in Fig. 2. Clear apoptotic bodies (in early, late and very late stages) and condensed chromatin were seen in PA1 treated cells (both with **C**_**1**_ and Doxorubicin). All the results are displayed as Fig. 2. Whilst the CHO cells remained healthy after **C**_**1**_ treatment, there were still a few apoptotic bodies in Doxorubicin-treated ones, indicating safety of **C**_**1**_ over Doxorubicin (further verification required).

**Figure 3:**
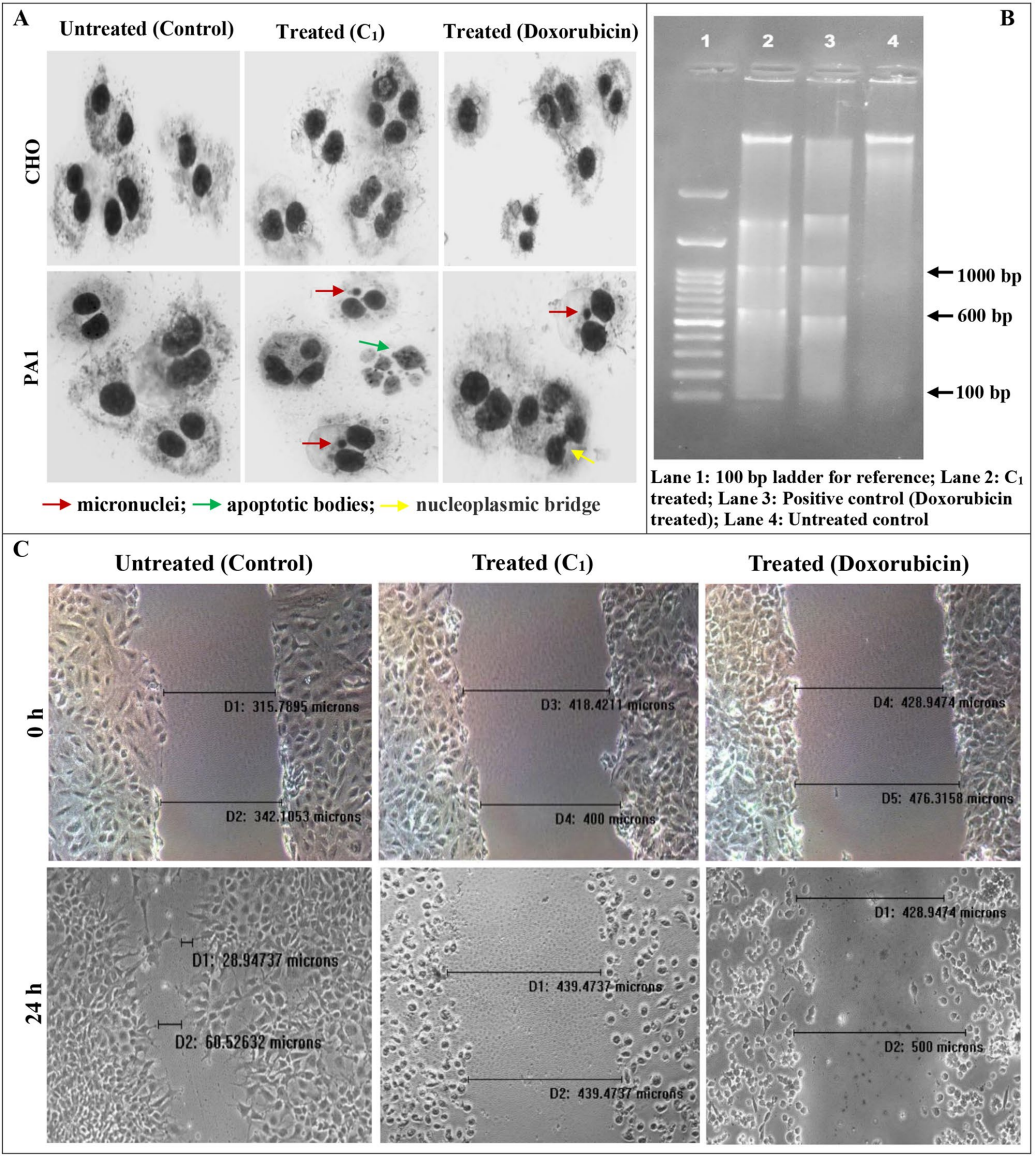
(**A**) clearly shows micronuclei (red arrowheads), apoptotic bodies (green arrowheads) and nucleoplasmic bridges (yellow arrowheads) for the **C**_**1**_ and Doxorubicin treated cells which are not visible in untreated cells either for CHO or PA1 cell lines. Clear DNA fragmentation is seen for the cells [PA1] (treated with either **C**_**1**_/Doxorubicin) to direct apoptotic cell death (**B**). Cell migrations were clearly inhibited for **C**_**1**_/Doxorubicin treated PA1 cells [Distance migrated 7.5 and 2.4% respectively] as against their untreated controls [86.4%] (**C**).

When clear micronuclei, apoptotic bodies and nucleoplasmic bridges are formed when **C**_**1**_ was introduced to PA1 cells, the same was not evident in the CHO cells (Fig. 3A). The frequency of occurrence of cells with micronuclei was found to be 0.0035 ± 0.0007, 0.0035 ± 0.0007 and 0.005 ± 0.001 for CHO and 0.019 ± 0.0021, 0.097 ± 0.0042 and 0.098 ± 0.0042 for PA1 cells in that order, clearly signifying genotoxic effects of **C**_**1**_ to the latter while proving safe to non-target cell populations. NDI was calculated as a measure of cell proliferation and it was found to be 2.331 ± 0.084, 2.269 ± 0.017 and 1.859 ± 0.045 for CHO and 2.557 ± 0.005, 1.795 ± 0.004 and 1.809 ± 0.011 for PA1 cells, clearly signifying genotoxic effect of **C**_**1**_ to the latter cell type. Clear DNA laddering was induced by **C**_**1**_ as well as Doxorubicin to PA1 cells but not in untreated ones (Fig. 3B), hinting apoptosis as its mechanism of action. When the distance migrated by PA1 cells between 0 and 24th h treatment period in **C**_**1**_-induced cell lines was found to be 7.5% and 2.4% for Doxorubicin-induced ones, the untreated ones were found to migrate with least resistance (86.4%), indicating anti-migratory effect of **C**_**1**_ (Fig. 3C).

**Figure 4:**
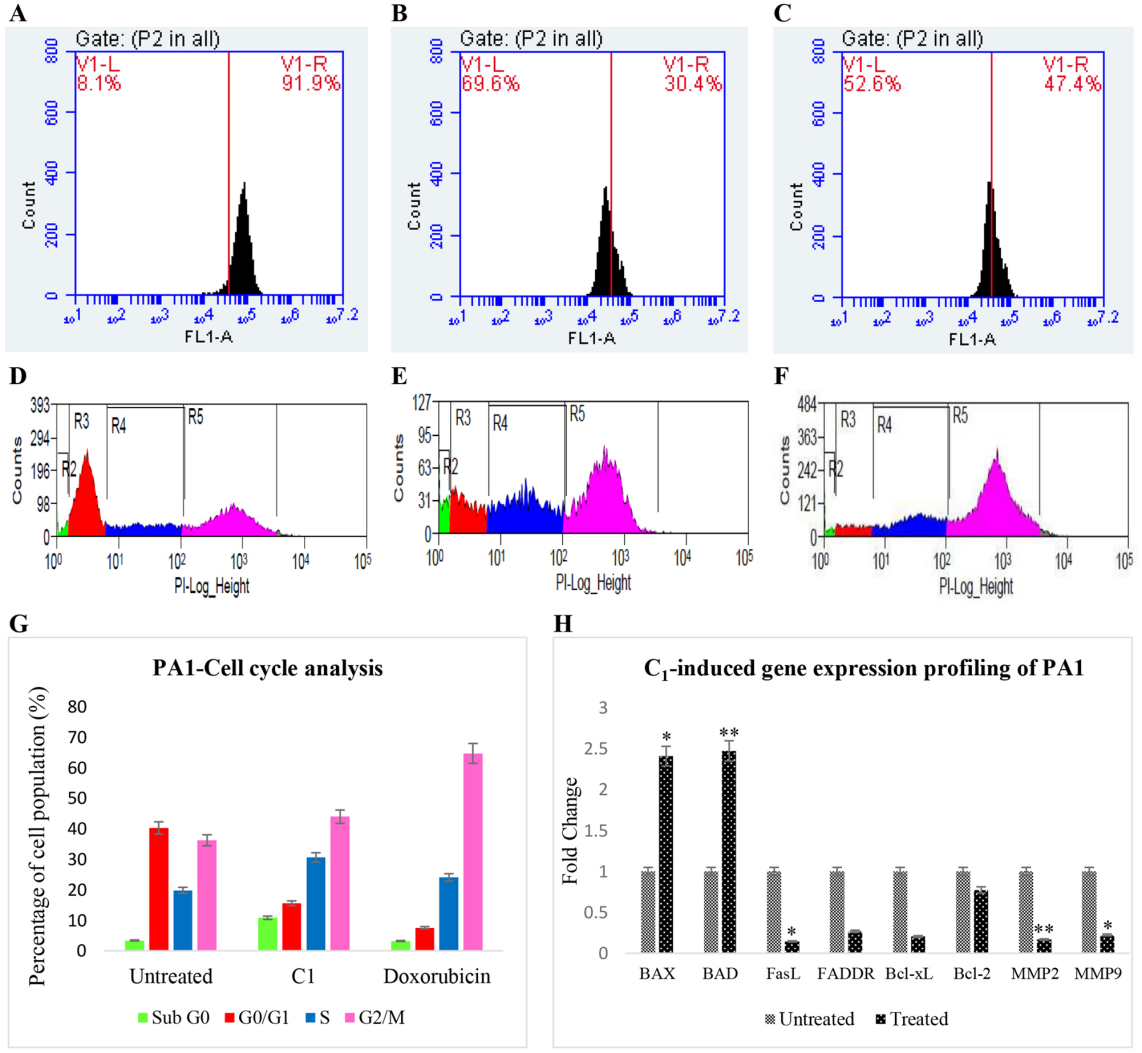
Rhodamine 123 dye fluorescence as a measure of Mitochondrial Membrane Potential (MMP) was determined in intact and distorted mitochondria. A clear reduction in the fluorescence was observed for the **C**_**1**_ treated PA1 cells [**B**-69.6%] from the untreated controls [**A**-8.1%]. Doxorubucin also shows a reduction in fluorescence [**C**-52.6%], however not as efficient as **C**_**1**_-induced mitophagy followed by MMP depolarization. The cells piled up at S as well G2/M phases for the **C**_**1**_ treated groups (**E** and **G**), whereas doxorubicin is a known G2/M phase arrester (**F** and **G**) as against their untreated controls where cells were distributed across all phases (**D** and **G**). Clear up-regulation of mitochondrial-associated factors, BAX and BAD, down-regulation of death receptor-related FasL and FADDR and anti-apoptotic gene [Bcl-xL, Bcl-2, MMP2 and MMP9] expressions hint intrinsic apoptotic route for **C**_**1**_ treated PA1 cells as against their untreated counterparts (**H**) (* *p* ˂ 0.05, ** *p* ˂ 0.005).

Organelle-specific changes during **C**_**1**_/Doxorubicin treatment was also analyzed by measuring fluorescent shifts in flow cytometer that indicate change in the Mitochondrial Membrane Potential (MMP). Clear MMP shift was noted for **C**_**1**_**-**introduced cells, which was higher (69.6%) than that of Doxorubicin-induced ones (52.6% of cells shifted) as against the untreated counterparts (only 8.1% showed reduced fluorescence). This evidenced a fair MMP disturbance in the treated ones. Similar to the aforementioned results, **C**_**1**_ pushed the cells to get piled up both at S and G2/M phases, indicating dual specificities. Results of the gene expression studies also quite reveal specific mRNA profiling. It was noted that **C**_**1**_ increased the expression of apoptotic genes, BAX and BAD and down-regulated the cell survival and metastatic genes, Bcl-xL, Bcl-2, MMP2 and MMP9, in comparison to the untreated controls (Fig. 4), to cause plausible cell death in the treated group.

### Bioinformatics

**Figure 5:**
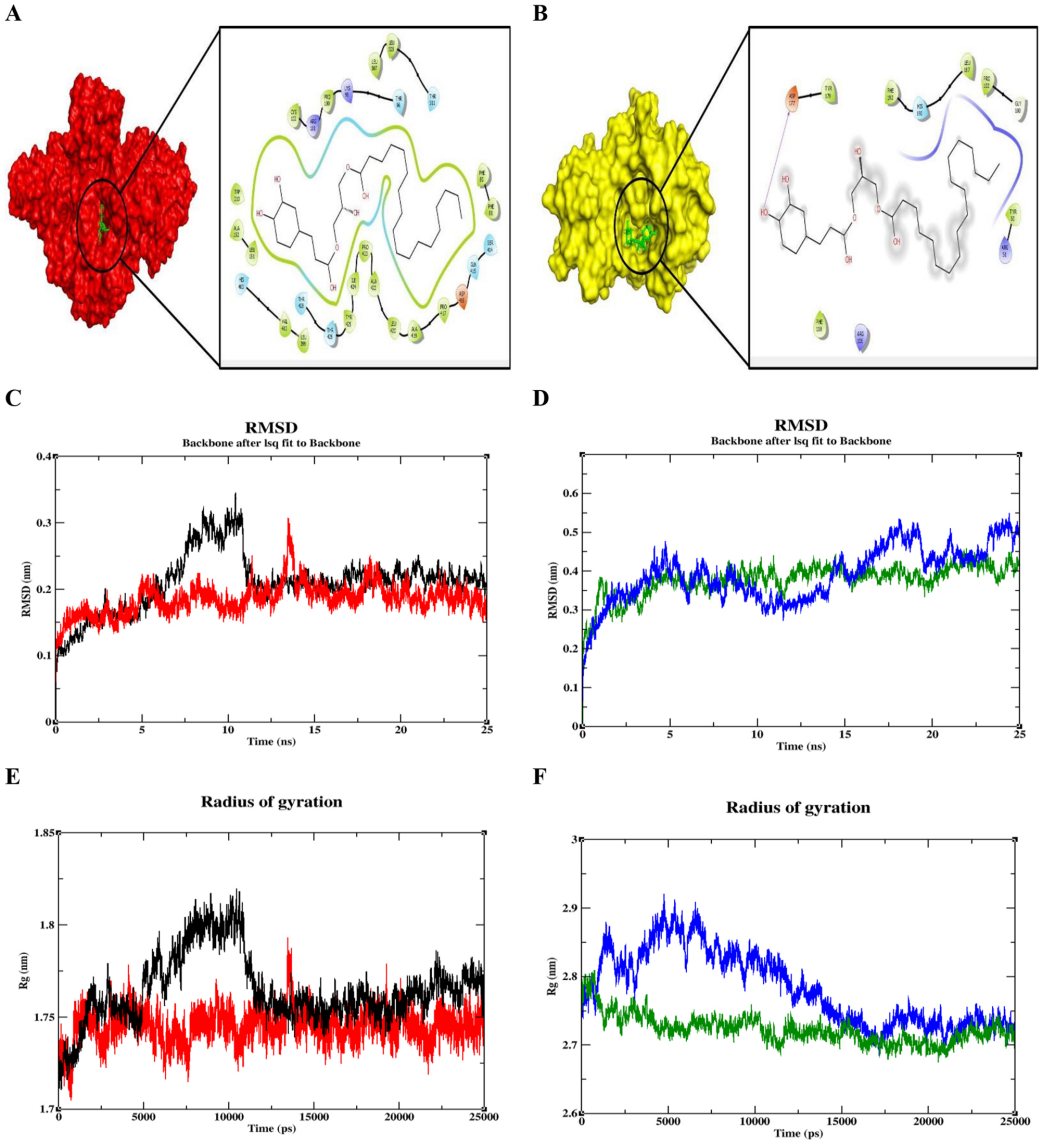
Molecular docking analysis of **C**_**1**_ (green) with MMP2 (red) (**A**) and MMP9 (yellow) (**B**) (Schrödinger, NY, 2018) indicate binding of **C**_**1**_ to (> 20 aminoacids of) MMP2-PEX region and (> 10 aminoacids of) MMP9. Molecular Dynamic simulations for **C**_**1**_ was performed and the stability with MMP2 (**C**) and MMP9 (**D**) was ascertained for 25 ns determine the extent of stability of C_1_-target complex. Radius of Gyrations (Rg) was carried out to predict the levels of compactness for **C**_**1**_-target complex (MMP2) (**E**) and MMP9 (**F**). Color scheme: native MMP2 (Black); **C**_**1**_-MMP2 complex (red); native MMP9 (blue) and **C**_**1**_-MMP9 complex (green).

Data for oral toxicity predictions, physicochemical and PK/PD properties of **C**_**1**_ are given as Fig. S7. The number of rotatable bonds exceeds 20, indicating **C**_**1**_ to be flexible and proficient to interactions with specific binding pockets. **C**_**1**_ is mentioned to have no AMES toxicity (a routine assay to test the mutagenicity of a drug), which is reassured by the negative results obtained for genotoxicity assays performed in normal CHO cell lines (Table S1). Target prediction for **C**_**1**_ assigned a maximal probability score to Hemopexin-like repeat (PEX) domain of MMP2 and a 67 kDa MMP9, both of which are Matrix MetalloProteinases, whose function is to dissolve the extracellular matrix and help in cell migration and metastasis. These two targets have a few interacting proteins in common such as TIMP (1, 2 and 3), STAT 3, VEGF A, SRC, DCN, MMP 10 and CD 44 (Fig. S8) and all are supposed to be plum targets for cancer therapies. **C**_**1**_ binds with docking scores of − 9.25 and − 9.30 kcal/mol with the targets, MMP2 and MMP9 respectively, which signifies least binding energies (Table S3) and it also shows that the binding of the ligand with both MMP2 and MMP9 proteins are almost similar. Further, the number of interacting amino acids of the proteins was visualized using Schrodinger Maestro and the ligand was shown to interact with MMP2 and MMP9 proteins with 28 and 11 amino acids respectively (A list of interacting aminoacids are also given in the Supplementary Table S3). Trajectories obtained from the molecular dynamics simulations were analyzed to understand protein deviation and change in compactness of the protein both in the native and target-bound forms. Overall deviations of the native MMP2 and MMP2-**C**_**1**_ ligand complex were found to be between ~ 0.1 and ~ 0.35 nm respectively and the complex showed lesser and stable deviation throughout the simulation and was almost same like the native MMP2. On the other hand, the overall Root Mean Square Deviations (RMSD) of the native MMP9 and MMP9-ligand complex showed deviations between ~ 0.1 and ~ 0.55 nm. Similar to MMP2-**C**_**1**_ complex, MMP9-ligand too showed lesser deviation to the native MMP9. Even the slightest deviations observed were found to be minimized and got stabilized to show convergence at 25 ns (Fig. 5). With promising results, the Radius of gyration (R_*g*_) was analyzed to understand changes in compactness of the protein. Overall R_*g*_ of native MMP2 and MMP2-**C**_**1**_ complex was found to be between ~ 1.7 and ~ 1.82 nm and MMP2-ligand complex showed lesser and stable deviation and merged with native MMP2. Overall R_*g*_ of native MMP9 and MMP9-**C**_**1**_ complex was found to be between ~ 2.66 and ~ 2.92 nm, suggestive of compactness and stability of the target-**C**_**1**_ complex with the native proteins (Fig. 5).

## Discussion

This paper describes in detail the isolation and structural elucidation of a novel compound, **C**_**1**_ isolated from the seagrass *Cymodocea serrulata,* which was identified as an ester of *trans*-3,4-dihydroxy cinnamic acid and linoleic acid connected to glycerol [IUPAC name: [3-(*E*-3,4-dihydroxycinnamaoyloxyl)-2-hydroxypropyl 9Z, 12Z-octadeca-9, 12-dienoate]. Clear anticancer properties were exhibited by **C**_**1**_ in both lung (A549) (data not shown herein) and ovarian cancer (PA1) cell lines with an IC_50_ value of 5.8 μM, which is lesser than the reference standard, Doxorubicin (8.6 μM). **C**_**1**_ absolutely caused no mortalities to non-cancerous Chinese Hamster Ovarian (CHO) cell lines even at 232. 25 μM i.e., 40 times higher the IC_50_ that was used for PA1 cells, pinpointing its safety to non-target cell population. The following key properties were detected for **C**_**1**_ on PA1 cells: formation of apoptotic bodies (early, late and very late stages), nuclear condensation, DNA fragmentation, inhibition to cellular migration, disturbance in MMP, piling up of cells both at S and G2/M phases, increased expression of pro-apoptotic genes [BAX and BAD] and down-regulation of cell survival genes [Bcl-xL, Bcl-2, MMP2 and MMP9. As mentioned above, **C**_**1**_ possessed dual (S and G2/M) cell cycle specificities in PA1 population. Generally speaking, there have certainly been not more than 5 of previously known molecules with dual arresting efficacies, such as a Cdc25 phosphatase inhibitor^25^, Gatifloxacin^26^, a topoisomerase poison^27^ and a HIV-1 viral protein R arresting G2/M via S phase-dependent mechanism^28^. Of course, all these compounds suggested more than a single route to kill the cells. In the current work, with the pulled down expression of FasL and FADDR, one could certainly exclude the option of extrinsic pathway for apoptosis. Additional to this, BAX and BAD upregulation could be very much linked to organelle-specific (mitophagy-mediated) cell death; all these outcomes indicate intrinsic apoptotic cascade. It has been shown that Matrix Metalloproteinases (MMPs) are expressed during G2/M transition and any arrest during this phase is known to downregulate the production of these secretory proteins in particular, gelatinases like MMP2^29^ which can be well related to the results of this investigation.

Despite the fact that **C**_**1**_ comes well under Toxicity Class: 5, it violates Lipinski’s filter by one parameter i.e., molecular weight greater than 500 Da; however, the number of hydrogen bond acceptors-7, donors-3 and the calculated MLog *P*_o/w_ (3.76) values still comply with Lipinski’s Ro5 for safe testing in in vivo models. Using in silico tools, it was identified that **C**_**1**_ binds to a variety of MMPs, out of which the top two in the list (MMP2 and MMP9) were chosen for doing molecular dynamic simulation testing. It is well known that MMPs are known targets in cancer therapies because of their known roles in cancer cell migration and invasion, mainly by dissolving extracellular matrix as mentioned in “Introduction” section. Reproductive cells in particular, ovaries, bank on MMPs, specifically type-2 and 9 as ovulation approaches and during luteolysis^30,31^. An interesting possibility for MMP activation during leuteolysis was proposed by Endo et al.^32^ who reported that collagenases are known to be activated by hypochloride and chloramines. These molecules can be generated by macrophages and neturophil peroxidases which utilize hydrogen peroxide, whose levels augment in the corpus luteum than other tissues upon luteolyis. Hence gelatinases in particular, have pivotal roles in ovulation and luteolysis, and any dysregulated MMP signaling can induce cancer. All MMPs have, in common, a signal peptide, a propeptide, a zinc binding domain, a hinge region. In addition to these, for MMP2 and 9, there are fibronectin-like repeats, and finally a hemopexin (PEX)-like domain located at their C-terminus. MMP2 and 9 are classified as gelatinases A and B respectively in the entire MMP family because only these degrade gelatin with high specificities. They are also known to use Collagen type IV of the extracellular matrix as their substrate. Despite design of drugs for the purpose of knocking down MMPs, many fail entry to clinical trials because all such drugs target the conserved catalytic domain of the protein^33,34^. Therefore, there occurred a paradigm shift from the point of view of targeting conserved regions to the less conserved non-catalytic functional groups, the PEX region. Out of the two gelatinases, **C**_**1**_ binds to the “most-sought after” PEX region of MMP2 and to an unknown site of MMP9. Least docking scores and binding energy indicate favorable energy requirements for target-**C**_**1**_ binding to MMP2-PEX region. RMSD and R_*g*_ studies also specify that there is compactness of “**C**_**1**_-target complex” with much less deviations from their native counterparts.

The main activation route of MMP2, is by the formation of “MMP2-MT1-MMP-TIMP 2” complex (Membrane Tethered Membrane- Type 1; Tissue Inhibitor of Matrix Metalloproteinases 2)^35,36^. When the N-terminal domain of MMP2 binds to and inhibits MT1-MMP, the C terminal binds to the PEX domain forming an active tertiary complex. Hence any blocking that may happen in the PEX domain may have significant roles in hampering MMP2 activation. Though much is known on the implication of PEX in MMP2, not many reports relating MMP2 to PEX-inhibitors^37^ are extant, as against many with MMP9^38–41^. On the whole, there are numerous inhibitors for MMPs and different classes of drugs are in use (Hydroxamate-based first generation broad spectral drugs, Marimastat, Ilomastat and batimastat, followed by new generation thiol based inhibitors, Rebimastat, Tanomasta). As indicated before, all these did not make it to the markets even after clearing clinical trials due to lack of specificities because they all target the conserved catalytic site of MMPs.

A few cinnamoyl derivatives were also examined in the past as possible MMP-2 inhibitors but their specificity to PEX region is not explored^42,43^. Cinnamoyl derivatives, like Cipralphelin^44^, Iotrochamides A and B^45^ are also isolated from the marine environment; however, they are not being evaluated as MMP inhibitors. Cinnamoyl derivatives, with an aromatic side chain, commonly are known to demonstrate elevated inhibitory activities to MMPs, possibly because of enhanced interaction with the hydrophobic regions of the amino acid residues. Emphasis was laid on the fact that increasing the conjugate system between an aromatic ring and an ester will either keep up or improve activities. Also, when the side chain was aliphatic as the length increased, the inhibitory activity of the compounds to MMP-2 was stronger^43^. So, with the available information on the chemically favorable structural requirements as mentioned in the research reports cited above, and the results of in vitro and in silico of the current investigation, **C**_**1**_ being a cinnamoyl-based compound with a long aliphatic chain containing an ester can be best evaluated in in vivo models. Results of the molecular dynamic simulation studies reveal that the MMP2-PEX-**C**_**1**_ complex is stable throughout the simulation period of 25 ns and in fact, showed lesser deviations to the native form.

## Conclusion

In the current investigation, it was understood that **C**_**1**_ has the following properties: (1) antiproliferative activity to PA1 cells as directed by intrinsic apoptotic route and regulating mRNA expression to that effect; (2) obeys 4 of the Lipinsky Ro5 and get aligned under Class: V; and (3) binds to the least explored PEX-region of MMP2 with high compactness and stability. Therefore, it is suggested that **C**_**1**_ being novel chemically may also possess novel bioactivities by being a specific target for PEX-domain of MMP2, to possibly inhibit a panel of cancer cells as well. Thorough molecular and toxicity studies, in vivo testing and convenient semi-synthesis of **C**_**1**_ could turn this unexplored molecule to a novel Gelatinase A-PEX inhibitor.

## Supplementary Information

Supplementary Information.
