# Supplementary material for: Inhibition of MMP2-PEX by a novel ester of dihydroxy cinnamic and linoleic acid from the seagrass Cymodocea serrulata
Source: Sci Rep. 2021 Jun 1;11:11451. doi: 10.1038/s41598-021-90845-9 (PMC8169913; doi:10.1038/s41598-021-90845-9)
Supplement: Supplementary file 1 — Supplementary Information. [file 41598_2021_90845_MOESM1_ESM.pdf]

Inhibition of MMP2-PEX by a novel ester of dihydroxy cinnamic and linoleic acid from the seagrass *Cymodocea serrulata*

[1] V. S. Christina, [2] R. Lakshmi Sundaram, *[3] V. Sivamurugan, [4] D. Thirumal Kumar, [2] C. D. Mohanapriya, [1] V. L. Shailaja, [5] S. P. Thyagarajan, [6] C. George Priya Doss and *[1]K. Mary Elizabeth Gnanambal

[1]Department of Biotechnology, Faculty of Biomedical Sciences and Technology a, SRI RAMACHANDRA Institute of Higher Education and Research (SRIHER), Deemed to be University (DU), Porur, Chennai, Tamil Nadu - 600 116, India

[2]Central Research Facility (CRF), SRI RAMACHANDRA Institute of Higher Education and Research (SRIHER), Deemed to be University (DU), Porur, Chennai, Tamil Nadu - 600 116, India

[3]PG & Research Department of Chemistry, Pachaiyappa's College, Chennai, Tamil Nadu – 600 030, India

[4]Meenakshi Academy of Higher Education and Research, Chennai, Tamil Nadu – 600 078, India

[5]Chancellor, Avinashilingam Institute for Home Science and Higher Education for Women (Deemed University), Coimbatore, Tamil Nadu - 641 043, India

[6]Department of Integrative Biology, School of Biosciences and Technology, Vellore Institute of Technology, Vellore, Tamil Nadu – 632014, India

***[1]Correspondence: drelizabethrajesh@sriramachandra.edu.in**

***[3]Correspondence: sivaatnus@gmail.com**

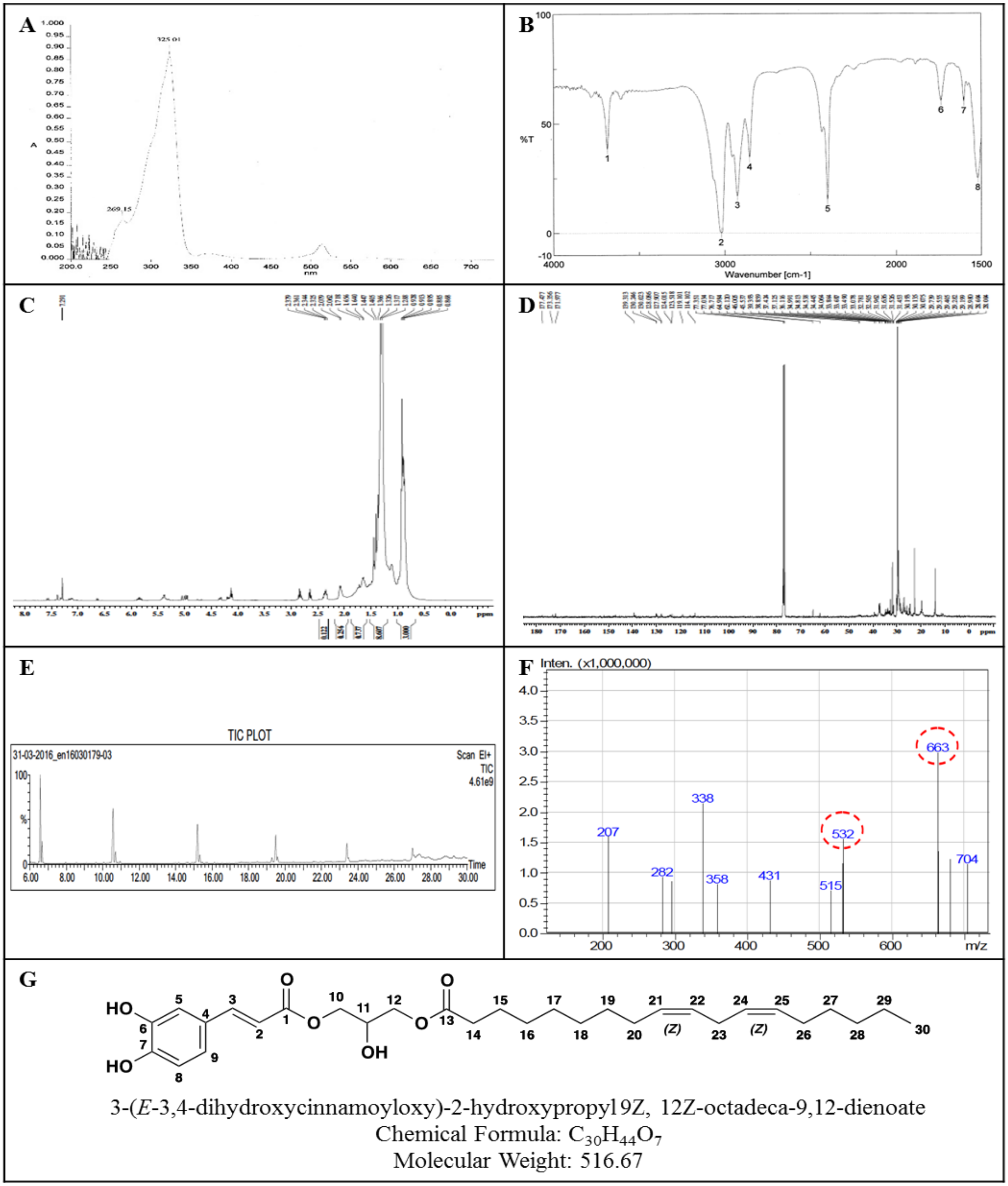

Figure S1. Spectral analyses of $\mathbf{C_1}$: Ultra violet/ Visible (UV/VIS) Spectroscopy (**A**), Fourier Transform-Infrared (FT-IR) Spectroscopy (**B**), ^{1}H Nuclear Magnetic Resonance (^{1}H-NMR) Spectroscopy (**C**), ^{13}C Nuclear Magnetic Resonance (^{13}C-NMR) Spectroscopy (**D**), Gas Chromatography-Mass Spectrometry (GC-MS) (**E**), Electron Spray Ionization-Mass Spectrometry (ESI-MS) (**F**) and the structural and molecular details of $\mathbf{C_1}$ (**G**).

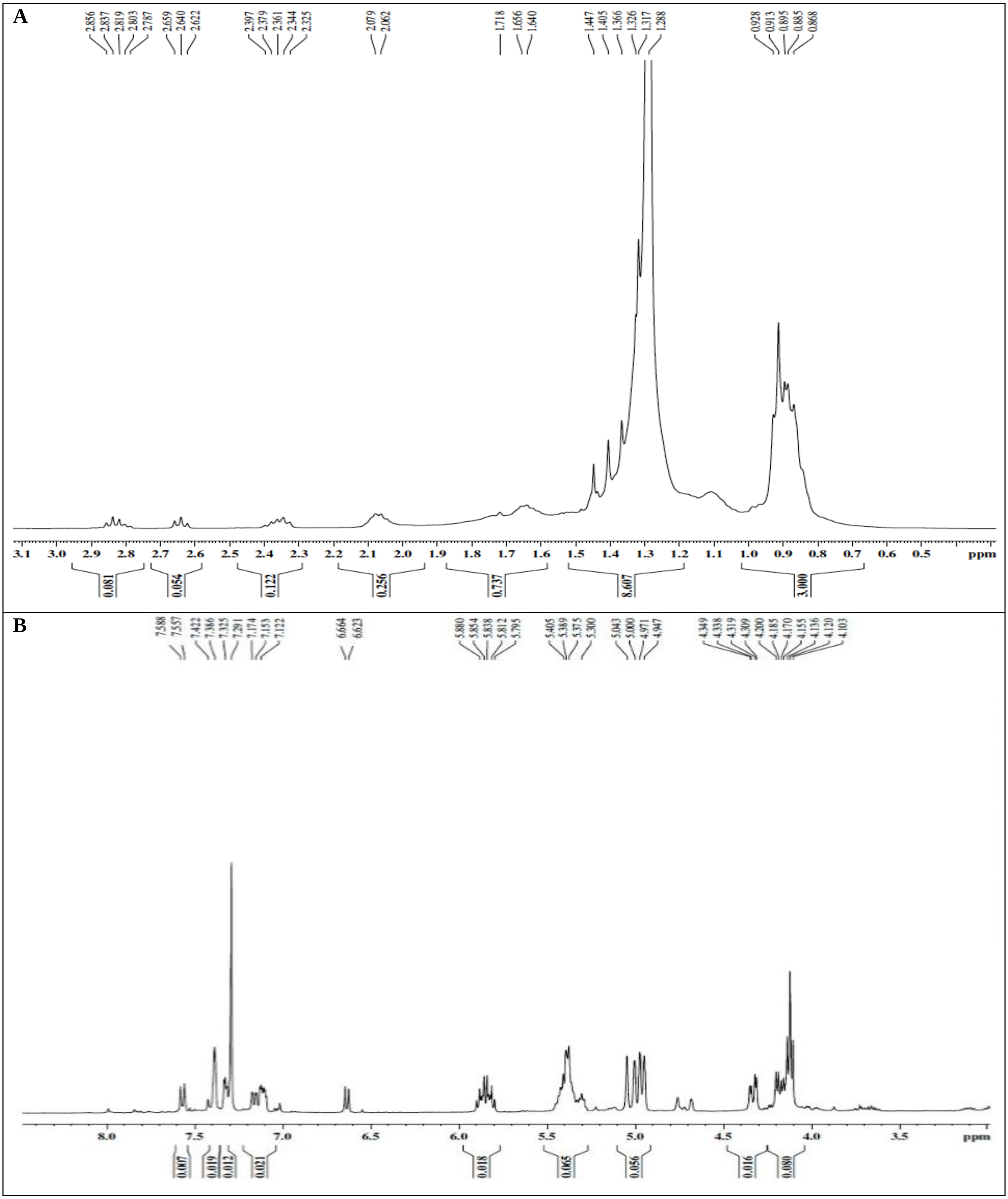

Figure S2. ^{1}H NMR spectrum of $\mathbf{C_1}$ (region 0.00-3.10 ppm) (**A**) and ^{1}H NMR expanded spectrum of $\mathbf{C_1}$(region 3.10 -8.00 ppm) (**B**).

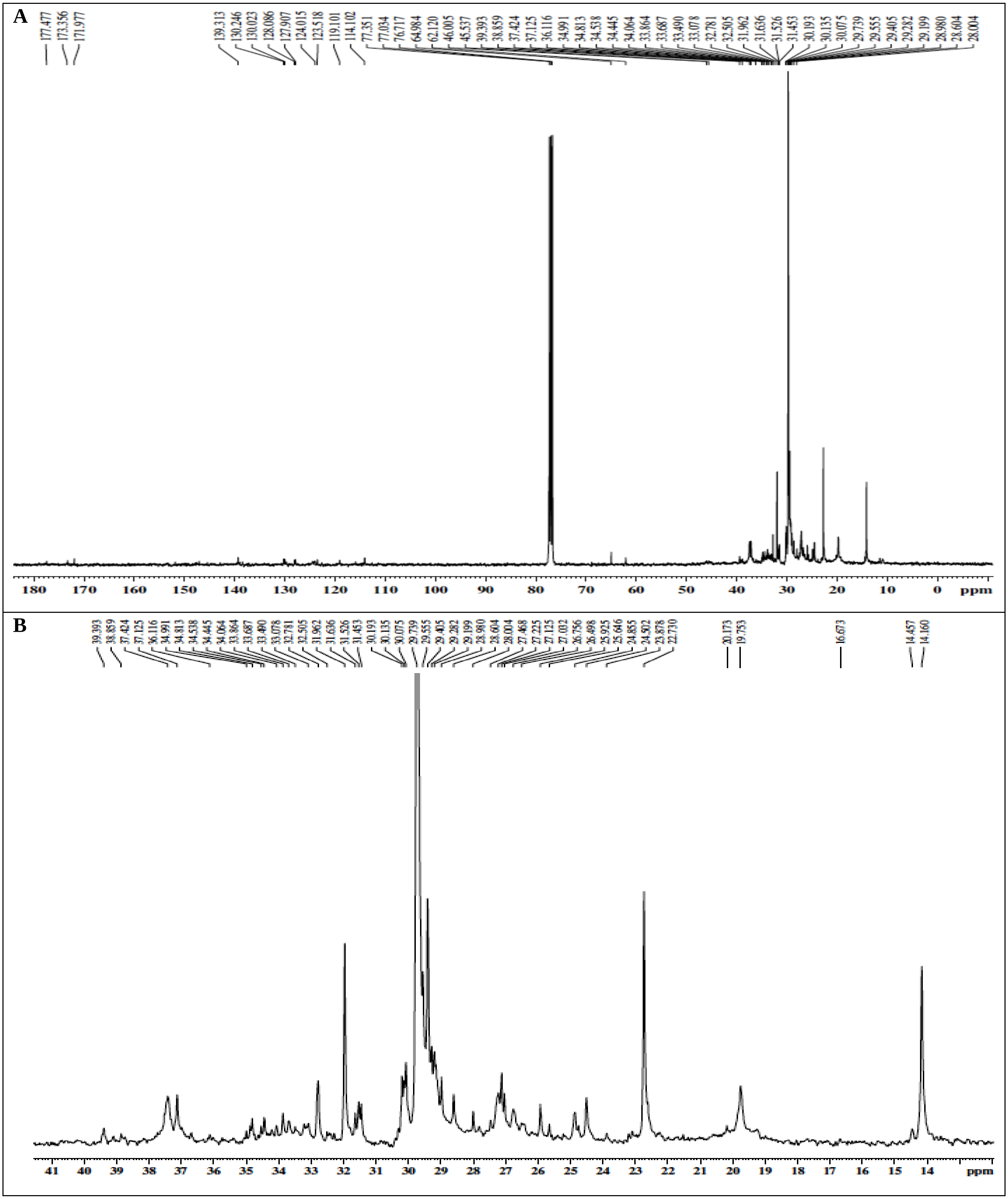

Figure S3. ^{13}C NMR spectrum of $\mathbf{C_1}$ (**A**) and its high field region (**B**).

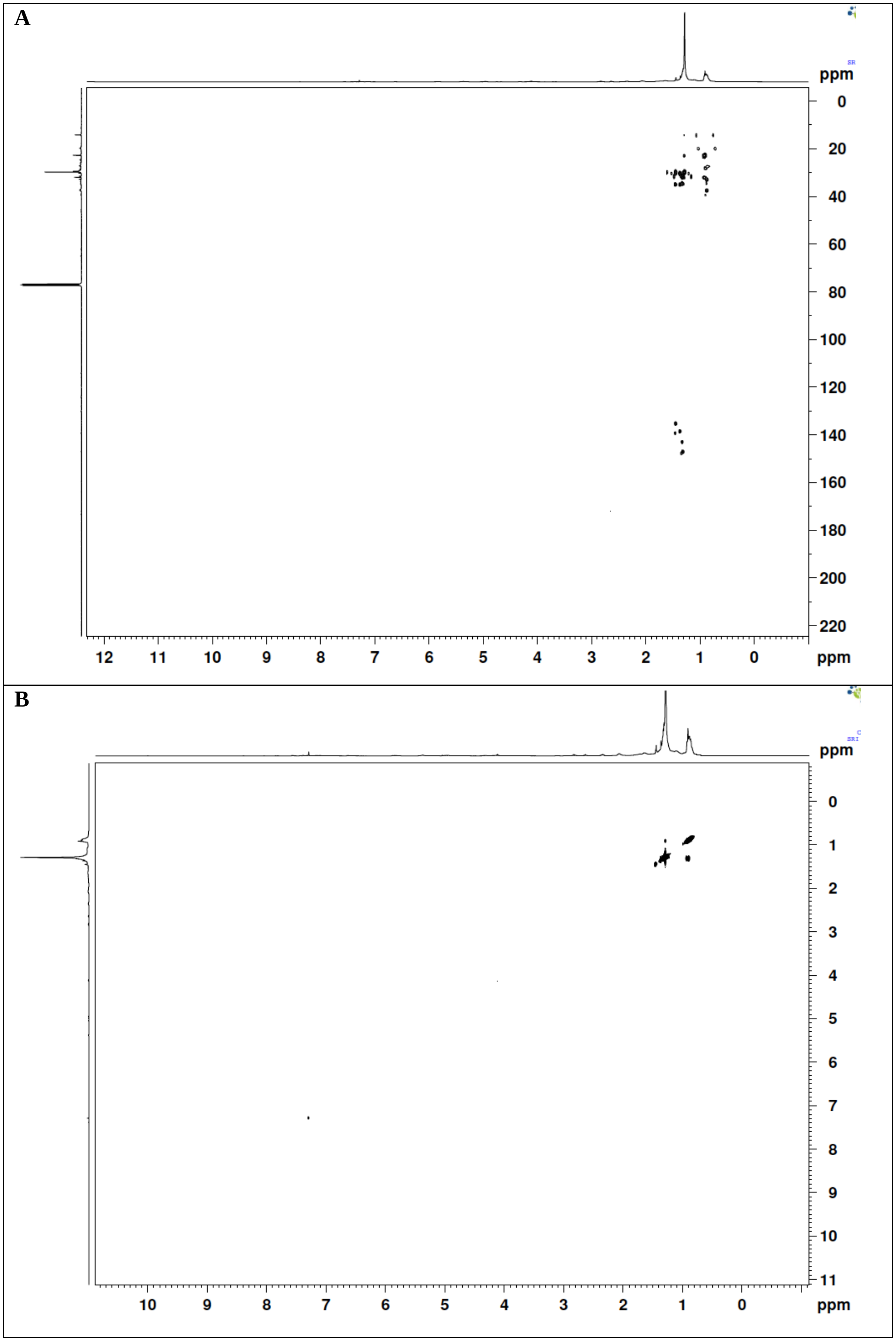

Figure S4. HMBC of **C_1** (**A**) and COSY of **C_1** (**B**).

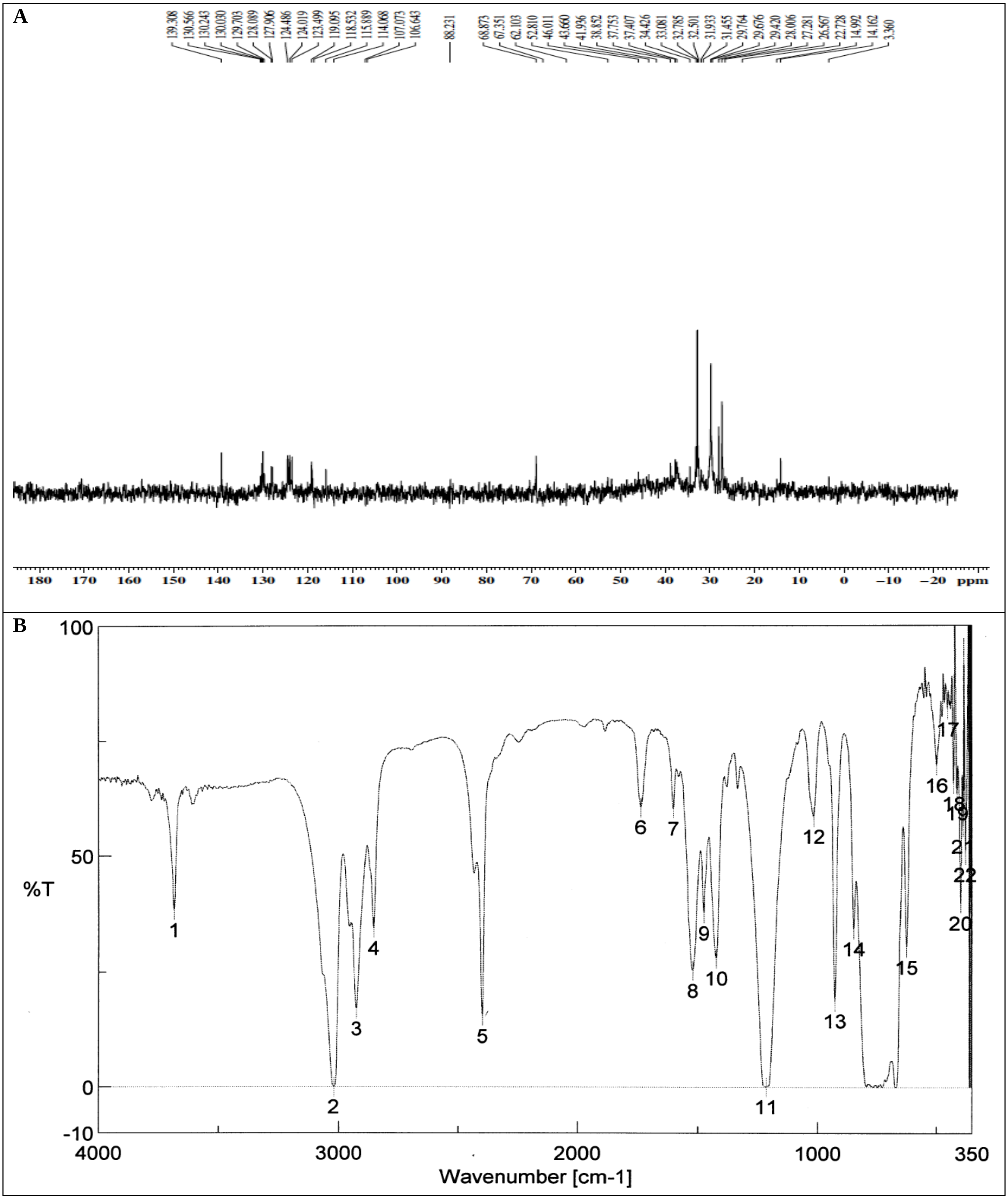

Figure S5. DEPT135 NMR spectrum of C_1 (**A**) and FT-IR spectrum of C_1 (**B**).

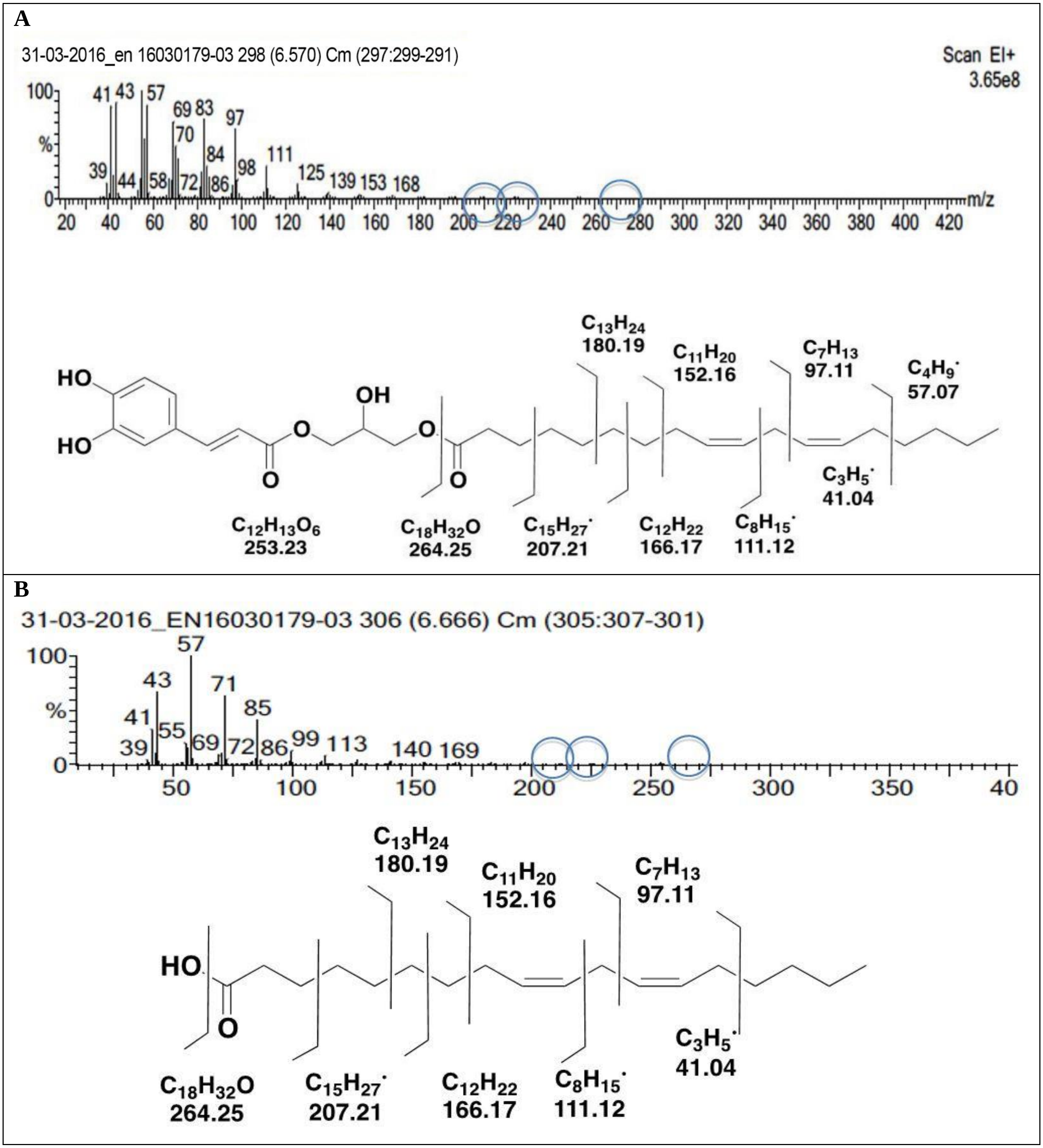

Figure S6. Mass spectrum of $\mathbf{C_1}$ analyzed in Gas Chromatography and the fragmentation patterns of the peaks appeared at 6.57 min (**A**) and 6.66 min (**B**).

A

Oral toxicity prediction results for input compound

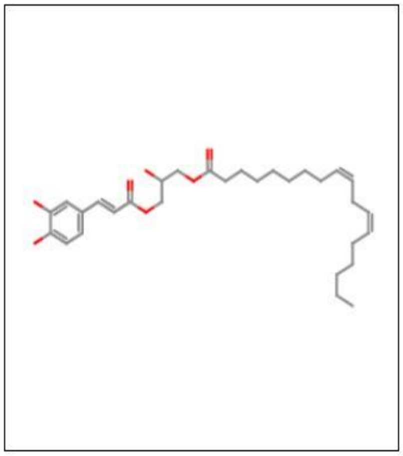

Predicted LD50: 5000mg/kg

Predicted Toxicity Class: 5

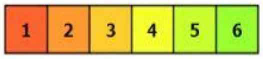

Average similarity: 69.63%

Prediction accuracy: 68.07%

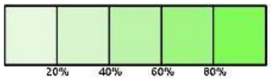

Name	CCCCCC=C/CC=C/CCC
Molweight	516.67
Number of hydrogen bond acceptors	7
Number of hydrogen bond donors	0
Number of atoms	37
Number of bonds	37
Number of rings	1
Number of rotable bonds	22
Total charge	0
Molecular Polar Surface Area	113.29

B

Molecule 1

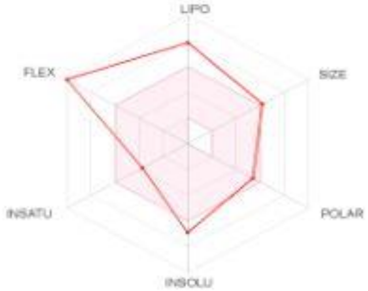

SMILES CCCCC/C=C\C/C=C\CCCCCCCC(=O)OCC(COC(=O)/C=C/c1ccc(c(c1)O)O)O

Physicochemical Properties	
Formula	C30H44O7
Molecular weight	516.67 g/mol
Num. heavy atoms	37
Num. arom. heavy atoms	6
Fraction Csp3	0.53
Num. rotatable bonds	22
Num. H-bond acceptors	7
Num. H-bond donors	3
Molar Refractivity	149.12
TPSA	113.29 Å²

Lipophilicity	
Log $P_{o/w}$ (iLOGP)	5.67
Log $P_{o/w}$ (XLOGP3)	8.26
Log $P_{o/w}$ (WLOGP)	6.26
Log $P_{o/w}$ (MLOGP)	3.76
Log $P_{o/w}$ (SILICOS-IT)	7.57
Consensus Log $P_{o/w}$	6.30

Water Solubility	
Log *S* (ESOL)	-6.92
Solubility	6.28e-05 mg/ml ; 1.22e-07 mol/l
Class	Poorly soluble
Log *S* (Ali)	-10.50
Solubility	1.63e-08 mg/ml ; 3.15e-11 mol/l
Class	Insoluble
Log *S* (SILICOS-IT)	-6.23
Solubility	3.07e-04 mg/ml ; 5.94e-07 mol/l
Class	Poorly soluble

Pharmacokinetics	
GI absorption	Low
BBB permeant	No
P-gp substrate	No
CYP1A2 inhibitor	No
CYP2C19 inhibitor	Yes
CYP2C9 inhibitor	Yes
CYP2D6 inhibitor	No
CYP3A4 inhibitor	No
Log K_p (skin permeation)	-3.59 cm/s

Druglikeness	
Lipinski	Yes; 1 violation: MW>500
Ghose	No; 4 violations: MW>480, WLOGP>5.6, MR>130, #atoms>70
Veber	No; 1 violation: Rotors>10
Egan	No; 1 violation: WLOGP>5.88
Muegge	No; 2 violations: XLOGP3>5, Rotors>15
Bioavailability Score	0.55

Medicinal Chemistry	
PAINS	1 alert: catechol_A
Brenk	4 alerts: catechol, isolated_alkene, michael_acceptor_1, more_than_2_esters
Leadlikeness	No; 3 violations: MW>350, Rotors>7, XLOGP3>3.5
Synthetic accessibility	5.01

Figure S7. Oral toxicity predictions – ProTox-II (above) and physicochemical and PK/PD properties of $\mathbf{C_1}$ using SwissADME (below).

A

Target	Uniprot ID	Gene code	ChEMBL ID	Probability	# sim. cmpds (3D / 2D)	Target Class
PEX	P08253	MMP2	CHEMBL333		1 / 3	Metallo Protease
67 kDa matrix metalloproteinase-9	P14780	MMP9	CHEMBL321		1 / 3	Metallo Protease
Macrophage metalloelastase	P39900	MMP12	CHEMBL4393		1 / 3	Metallo Protease
Collagenase 3	P45452	MMP13	CHEMBL280		1 / 3	Metallo Protease
22 kDa interstitial collagenase *(by homology)*	P03956	MMP1	CHEMBL332		1 / 3	Metallo Protease
Stromelysin-1 *(by homology)*	P08254	MMP3	CHEMBL283		1 / 3	Metallo Protease
Stromelysin-2 *(by homology)*	P09238	MMP10	CHEMBL4270		1 / 3	Metallo Protease
Matrix metalloproteinase-27 *(by homology)*	Q9H306	MMP27			1 / 3	Metallo Protease
Matrix metalloproteinase-20 *(by homology)*	O60882	MMP20	CHEMBL1938226		1 / 1	Enzyme
Protein kinase C gamma type *(by homology)*	P05129	PRKCG	CHEMBL2938		5 / 235	Ser_Thr Kinase
Protein kinase C beta type *(by homology)*	P05771	PRKCB	CHEMBL3045		5 / 235	Ser_Thr Kinase
Protein kinase C alpha type	P17252	PRKCA	CHEMBL299		5 / 235	Ser_Thr Kinase
Protein kinase C theta type *(by homology)*	Q04759	PRKCQ	CHEMBL3920		5 / 240	Ser_Thr Kinase
Protein kinase C delta type regulatory subunit *(by homology)*	Q05655	PRKCD	CHEMBL2996		5 / 240	Ser_Thr Kinase
Aldo-keto reductase family 1 member B10	O60218	AKR1B10	CHEMBL5983		0 / 21	Enzyme

B

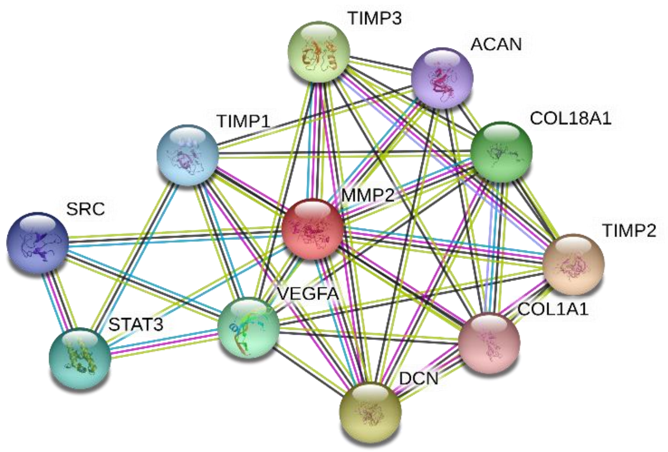

C

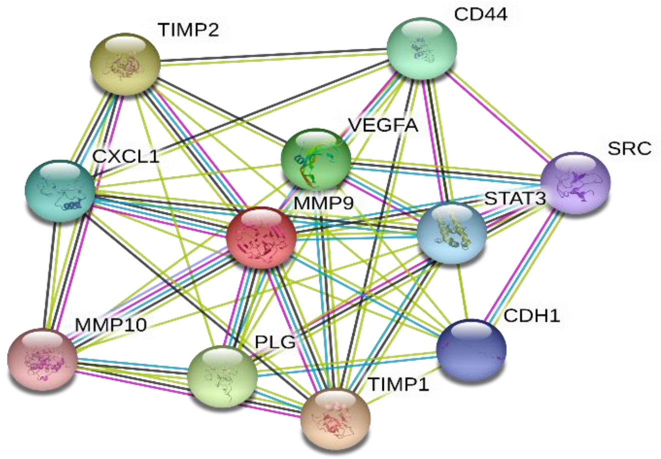

Figure S8. C_1 binds with maximal probability score to Hemopexin-like repeat (PEX) domain of MMP2 and to MMP9 as shown as **A** (SwissTatgetPrediction). The results of protein-protein interactions (STRING, version 11.0, 2019) indicate that MMP2 (**B**) and MMP9 (**C**) display commonalities in interacting with a huge number of proteins [TIMP (1, 2 and 3), STAT3, VEGFA, SRC, DCN, MMP10 and CD44], all of which play pivotal roles in cancer cascades, making both MMP2 and MMP9 plum targets for cancer metastasis inhibitions.

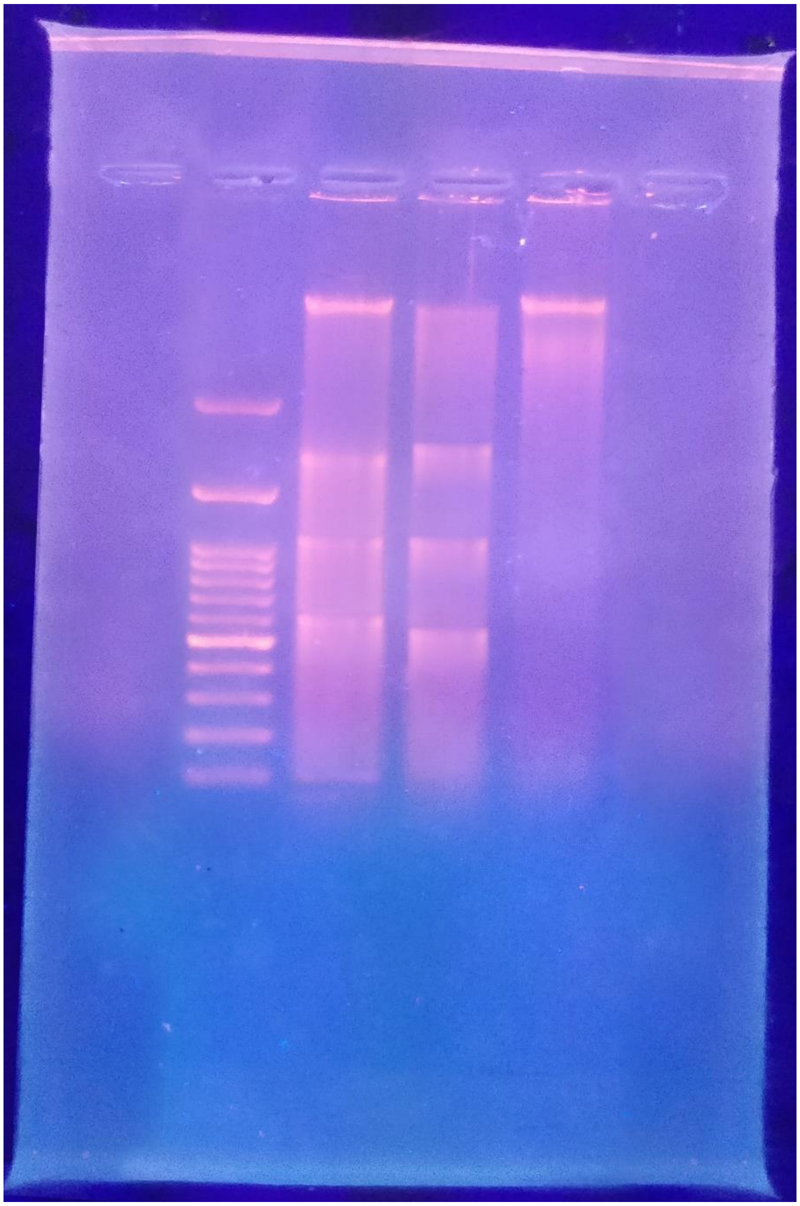

Figure S9. Full length gel picture of DNA fragmentation assay. Gray scale of this image showcasing only four lanes (cropped gel picture) is attached in Fig. 3B.

ADMET prediction using pkCSM tool		
**Property**	**Parameters**	**Values**
Absorption	Water solubility	-4.723
Absorption	Caco2 permeability	0.289
Absorption	Intestinal absorption (human)	72.601
Absorption	Skin Permeability	-2.728
Absorption	P-glycoprotein substrate	Yes
Absorption	P-glycoprotein I inhibitor	Yes
Absorption	P-glycoprotein II inhibitor	Yes
Distribution	VDss (human)	-0.372
Distribution	Fraction unbound (human)	0.078
Distribution	BBB permeability	-1.562
Distribution	CNS permeability	-3.245
Metabolism	CYP2D6 substrate	No
Metabolism	CYP3A4 substrate	Yes
Metabolism	CYP1A2 inhibitor	No
Metabolism	CYP2C19 inhibitor	No
Metabolism	CYP2C9 inhibitor	No
Metabolism	CYP2D6 inhibitor	No
Metabolism	CYP3A4 inhibitor	Yes
Excretion	Total Clearance	1.53
Excretion	Renal OCT2 substrate	No
Toxicity	AMES toxicity	No
Toxicity	Max. tolerated dose (human)	-0.33
Toxicity	hERG I inhibitor	No
Toxicity	hERG II inhibitor	Yes
Toxicity	Oral Rat Acute Toxicity (LD50)	2.992
Toxicity	Oral Rat Chronic Toxicity (LOAEL)	2.431
Toxicity	Hepatotoxicity	No
Toxicity	Skin Sensitisation	No
Toxicity	*T.pyriformis* toxicity	0.289
Toxicity	Minnow toxicity	-2.905

Table S1: Absorption, Distribution, Metabolism, Excretion and Toxicity (ADMET) analysis of $\mathbf{C_1}$ using pkCSM tool.

Gene	Orientation	Primer sequence 5'- 3'	Reference
GAPDH	F	CGACCACTTTGTCAAGCTCA	Elumalai et al., 2012
	R	CCCCTCTTCAAGGGGTCTAC	
BAX	F	GCTGGACATTGGACTTCCTC	
	R	CTCAGCCCATCTTCTTCCAG	
BAD	F	CCTCAGGCCTATGCAAAAAG	
	R	AAACCCAAAACTTCCGATGG	
FasL	F	CCATGTGAAGAGGGAGAAGC	
	R	AAGACAGTCCCCCTTGAGGT	
Bcl-xL	F	GGCTGGGATACTTTTGTGGA	
	R	AAGAGTGAGCCCAGCAGAAC	
Bcl-2	F	ATTGGGAAGTTCAAATCAGC	
	R	TGCATTCTTGGACGAGGG	
FADDR	F	AGATGAACCTGGTGGATGAC	
	R	AGGACGCTTCGGAGGTAGAT	
MMP-2	F	TTTCCATTCCGCTTCCAGGGCAC	Zhan et al., 2012
	R	TCGCACACCACATCTTTCCGTCACT	
MMP-9	F	CCTGCCAGTTTCCATTCATC	
	R	GCCATTCACGTCGTCCTTAT	

Table S2: Forward and Reverse sequences of primers for the genes used for the study.

Protein	Ligand	Docking Score	Glide Energy	Surrounding Amino Acids
MMP2 (1CK7)	$\mathbf{C_1}$	-9.258 kcal/mol	-56.776 kcal/mol	CYS102, ARG101, PRO100, LYS99, LEU507, LEU508, THR96, THR511, PHE80, PHE81, SER414, GLN415, ASP416, PRO417, ALA419, LEU420, ALA422, PRO423, ILE424, TYR425, THR426, THR428, LEU399, VAL400, HIS403, LEU191, ALA192, TRP213
MMP9 (5UE3)	$\mathbf{C_1}$	-9.307 kcal/mol	-50.948 kcal/mol	ASP177, TYR179, PHE192, HIS190, LEU187, PRO102, GLY100, TYR50, ARG51, ARG106, PHE110

Table S3: Docking details of MMP2 and MMP9 with $\mathbf{C_1}$.
